# Single stranded DNA annealing is a conserved activity of telomere resolvases

**DOI:** 10.1371/journal.pone.0246212

**Published:** 2021-02-04

**Authors:** Siobhan L. McGrath, Shu Hui Huang, Kerri Kobryn

**Affiliations:** Department of Microbiology & Immunology, College of Medicine, University of Saskatchewan, Saskatoon, Saskatchewan, Canada; Tulane University Health Sciences Center, UNITED STATES

## Abstract

Bacterial species of the genera *Agrobacterium* and *Borrelia* possess chromosomes terminated by hairpin telomeres. Replication produces dimeric replication intermediates fused via replicated telomere junctions. A specialized class of enzymes, referred to as telomere resolvases, promotes the resolution of the replicated intermediate into linear monomers terminated by hairpin telomeres. Telomere resolution is catalyzed via DNA cleavage and rejoining events mechanistically similar to those promoted by topoisomerase-IB and tyrosine recombinase enzymes. Examination of the borrelial telomere resolvase, ResT, revealed unanticipated multifunctionality; aside from its expected telomere resolution activity ResT possessed a singled-stranded DNA (ssDNA) annealing activity that extended to both naked ssDNA and ssDNA complexed with its cognate single-stranded DNA binding protein (SSB). At present, the role this DNA annealing activity plays *in vivo* remains unknown. We have demonstrated here that single-stranded DNA annealing is also a conserved property of the agrobacterial telomere resolvase, TelA. This activity in TelA similarly extends to both naked ssDNA and ssDNA bound by its cognate SSB. TelA’s annealing activity was shown to stem from the N-terminal domain; removal of this domain abolished annealing without affecting telomere resolution. Further, independent expression of the N-terminal domain of TelA produced a functional annealing protein. We suggest that the apparent conservation of annealing activity in two telomere resolvases, from distantly related bacterial species, implies a role for this activity in hairpin telomere metabolism. Our demonstration of the separation of the telomere resolution and annealing activities of TelA provides a platform for future experiments aimed at identifying the role DNA annealing performs *in vivo*.

## Introduction

**Fig 1:**
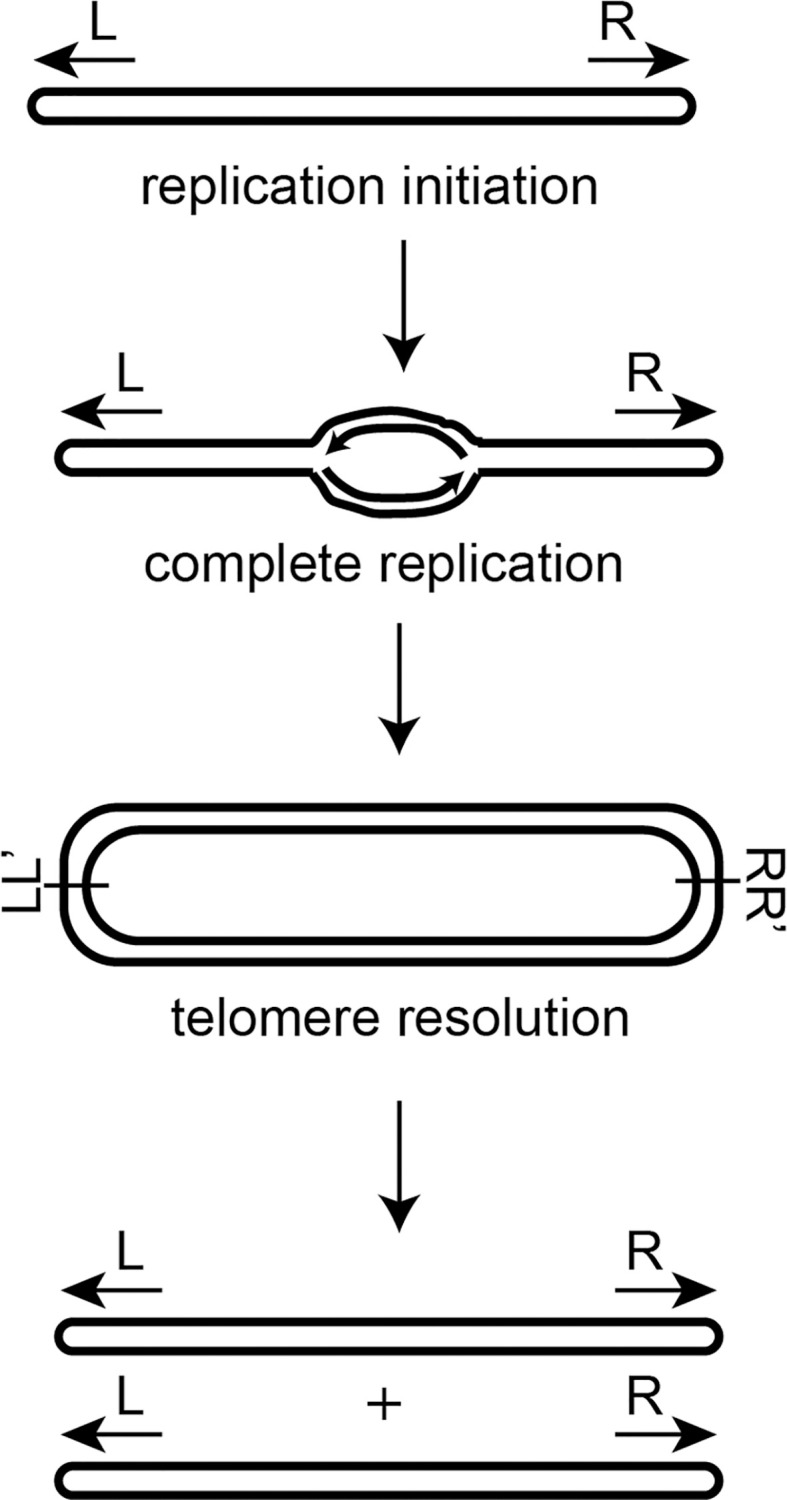
A model of bacterial linear DNA replication. Replication of the linear DNA molecule initiates at an internal origin and proceeds bidirectionally through the hairpin telomeres. This produces a circular dimer of replicated DNA fused at replicated telomere junctions containing inverted repeat symmetry (denoted at L/L’ and R/R’). The replication intermediate is then resolved into two linear replicons closed by hairpin ends through a process called telomere resolution.

In contrast to the majority of prokaryotes, a select few maintain linear replicons including chromosomes, plasmids or some combination of both. Organisms possessing linear DNAs require specialized strategies to bypass the twin issues of priming lagging strand synthesis at the termini—termed the end-replication problem, and protecting the DNA’s exposed ends from inappropriate fusion or degradation–the end-protection problem [1, 2]. Perhaps the conceptually simplest strategy implemented to circumvent these problems is the use of hairpin (hp) telomeres; structures that constitute a covalently closed DNA hairpin loop at the termini of the replicon to eliminate the free end and thus, the source of these problems [3]. Hairpin telomeres can be found in the *Borrelia* species [4–6], *Agrobacterium tumefaciens* biovar I strains [7], and various phage [8–10]. The prevailing model for bacterial linear replication comes from studies of *Borrelia burgdorferi* which suggest bi-directional replication from an internal origin that then proceeds through the hp telomeres [5, 11]. This produces a circular dimer intermediate joined at replicated telomere junctions (*rTel*) possessing inverted repeat symmetry. This dimer is then resolved into two linear replicons terminated by hp telomeres through a unique, two-step breakage and rejoining reaction called telomere resolution (Fig 1 & [12, 13]). Specialized enzymes called telomere resolvases are required to perform this function [14]. They promote telomere resolution via a catalytic domain and reaction chemistry displaying similarities to that of the topoisomerase-IB and tyrosine recombinase enzyme families.

Telomere resolvases from *Agrobacterium*, *Borrelia*, *Escherichia coli* phage N15, and *Klebsiella oxytoca* phage φKO2 have been studied *in vitro* at the biochemical and/or structural level [15–20]. In combination, this research reveals the diversity across this enzyme family in terms of size, domain composition, and even in the details of the mechanisms used to effect strand foldback into the hp conformation [15, 16, 21, 22]. However, the chemistry of the phosphoryl transfer appears to be conserved between different telomere resolvases by virtue of their shared catalytic domain. The borrelial telomere resolvase, ResT, and TelN from N15 phage have also been studied *in vivo*. ResT was shown to be essential [23]; however, conditional expression of ResT produced an unexpectedly complex phenotype wherein cells depleted of ResT did not filament (as would be expected in cells accumulating chromosome dimers) and instead ceased DNA replication [24]. This suggested ResT may interact directly or, indirectly, with *Borrelia’s* replication machinery. The telomere resolvase from N15 phage, TelN, was shown to not only to be needed for linear lysogen maintenance, but to be involved in establishing the substrate for lytic replication [25, 26]. Collectively, these *in vivo* studies suggested possible multifunctionality. Subsequent biochemical characterization of ResT revealed the borrelial telomere resolvase possessed single-stranded DNA annealing activity with naked DNA, as well as the ability to interact both physically and functionally with its cognate single-stranded DNA binding protein (SSB) to promote the annealing of SSB-complexed ssDNAs [27, 28]. This function is typical of recombination proteins that can promote RecA-dependent (*e*.*g*. RecO [29, 30]) and RecA-independent (*e*.*g*. λ beta [31, 32]) recombination, although the function of this annealing activity in ResT currently remains unknown.

No separation of function alleles currently exist to isolate ResT’s annealing and telomere resolution activities from one another. Due to the domain diversity across the telomere resolvase enzyme family, it was also unclear whether additional members would possess multifunctional properties similar to those observed in ResT. To investigate the potential multifunctionality among telomere resolvases, we sought to characterize an additional member of the telomere resolvase family. We selected TelA from *Agrobacterium tumefaciens* since its telomere resolution activity has already been demonstrated *in vitro* [20], it is the closest telomere resolvase to ResT in both size and sequence homology, and there are partial structural data available [16].

We report here that TelA possesses a single-stranded DNA annealing activity similar to that observed in ResT. TelA can anneal both naked ssDNA and ssDNA complexed with its cognate SSB. TelA also displays a limited strand exchange activity typical of annealing proteins. While the N-terminal domain of TelA was previously shown to be dispensable for telomere resolution [20], we demonstrate here that deletion of the N-terminal domain eliminates TelA’s annealing activity. The TelA N-terminal domain expressed as an independent protein recapitulated all the essential annealing and strand exchange activities displayed by the full length protein. Moreover, we show that a mutant of the telomere resolution active site nucleophile, TelA (Y405F), represents a separation of function mutant that only abolishes telomere resolution activity. Together, the N-terminal deletion mutant and the TelA (Y405F) highlight the physical and functional separation of the annealing and telomere resolution activities of a telomere resolvase.

## Materials and methods

### DNAs

All oligonucleotides and synthetic genes were purchased from Integrated DNA Technologies (IDT). The telomere resolvase, TelA (UniProtKB/Swiss-Prot: Q7CWV1), and SSB (UniProtKB/Swiss-Prot: Q8UF87.2) reading frames from *Agrobacterium tumefaciens* strain C58 were produced as synthetic genes codon-optimized for expression in *E*. *coli* flanked by NdeI/BamHI sites and blunt end cloned in pIDTSMART-AMP. The reading frames were excised by NdeI/BamHI digestion and cloned into pET15b digested with NdeI/BamHI to produce constructs with an N-terminal 6XHis tag for expression in *E*. *coli*. TelA (107–442) and TelA (1–106) were generated by PCR amplification from the TelA syngene plasmid using primers OGCB794/795 and OGCB876/877, respectively. A plasmid construct for overexpressing N-terminal His-tagged TelA (Y405F) was generated through site-directed mutagenesis of the TelA syngene plasmid using primers OGCB778/779. SSBΔC7 was generated by PCR amplification from the SSB syngene plasmid using primers OGCB796/797 followed by digestion with NdeI/BamHI and cloning into pET15b. These plasmids were all verified through DNA sequencing and archived in DH5α. These plasmids were transformed into Novagen’s Rosetta™(DE3)pLysS for expression and purification. Synthetic gene and oligonucleotide sequences are available in the Supplementary information as S1 and S2 sequence and S1 Table.

### Proteins

Expression of TelA was performed in 500 mL of LB broth containing 100 μg/mL ampicillin and 30 μg/mL chloramphenicol. A seed culture of the expression strain containing 100 μg/mL ampicillin, 30 μg/mL chloramphenicol, and 1% glucose was grown overnight at 37°C. The 500 mL culture was seeded 1 in 100 from the overnight culture of the expression strain and grown at 37°C to an A600nm of 0.4. The temperature was reduced to 24°C for 20 min followed by induction with 500 μM Isopropyl β-D-1-thiogalactopyranoside (IPTG). The culture was induced overnight at 24°C. Lysate was prepared from the pelleted cells by the freeze-thaw method previously described by [33]. The salt concentration of the lysate was adjusted to 0.5 M with Ni-load buffer (50 mM NaH2PO4, 10 mM imidazole, 10% glycerol) not containing NaCl and the lysate was loaded to a 10 mL Ni-NTA affinity column. The column was washed with 10 column volumes of 0.5 M NaCl Ni-wash buffer (50 mM NaH2PO4, 20 mM imidazole, 10% glycerol) and TelA was eluted with 15 mL of 0.5 M NaCl Ni- elution buffer (50 mM NaH2PO4, 400 mM imidazole, 10% glycerol) into 1 mL aliquots. Peak fractions were identified by sodium dodecyl sulfate (SDS) polyacrylamide gel electrophoresis (PAGE) and pooled. The combined Ni-elutions were diluted in HG buffer (25 mM HEPES [pH 7.6], 0.2 mM EDTA, 10% glycerol) containing no salt to reduce the salt concentration to 0.25 M NaCl and loaded to a 6 mL Heparin-Sepharose CL6B column (HS). The HS column was washed with 10 column volumes of 0.25 M NaCl HG and 2 column volumes of 0.35 M NaCl HG. TelA was eluted from the HS column with 9 mL of 0.5 M NaCl HG followed by 9 mL of 1.5 M NaCl HG in 1.5 mL fractions. Peak fractions from 1.5 M NaCl HG fractions were pooled and the protein concentration determined by using BioRad’s protein dye reagent (Bradford, 1976). TelA (Y405F) expression was performed as described for wildtype TelA.

For overexpression of TelA (107–442), the culture was induced at 27°C overnight with 250 μM IPTG. Lysate preparation, Ni-NTA affinity purification were performed as with wildtype TelA. The HS purification was performed similarly to the previously described protocol, however the column was eluted on a 14 mL linear gradient (0.25 M NaCl HG– 1.5 M NaCl HG). Peak fractions were identified by SDS-PAGE and tested for nuclease activity with the 3’-partial duplex substrate assembled from 5’-^32^P endlabelled OGCB666 and unlabeled OGCB692. Fractions containing the least amount of nuclease activity were pooled and the salt adjusted to 0.5 M NaCl. Pooled elutions were loaded to a 3 mL hydroxyapatite (HAP) column equilibrated with 0.5 M NaCl HG. The column was washed with 10 column volumes of 0.5 M NaCl HG + 10 mM sodium phosphate (NaPi) buffer. TelA (107–442) was eluted with a 9 mL linear gradient (HG 0.5M NaCl + 10 mM NaPi–HG 0.5M NaCl + 0.3M NaPi). TelA (1–106) was overexpressed and purified in a similar manner to wildtype TelA with a few alterations. TelA (1–106) was induced with 125 μM IPTG. The 10 mL HS column was eluted with 1 column volume of 0.5 M NaCl HG followed by one column volume of 0.8 M NaCl HG.

The overexpression and lysate preparation of *Agrobacterium tumefaciens* SSB and SSBΔC7 were performed in a similar manner to wildtype TelA. SSB and SSBΔC7 were induced with 125 μM and 500 μM IPTG, respectively. Following lysate preparation, the salt concentration of the SSB/SSBΔC7 lysate was adjusted to 0.8 M NaCl with Ni-load buffer lacking NaCl. SSB lysate was loaded to a 6 mL Ni-NTA column and SSBΔC7 lysate was loaded to a 30 mL Ni-NTA column. Both columns were washed with 10 column volumes of 0.8 M NaCl Ni-NTA wash buffer, and SSB and SSBΔC7 were eluted with 0.8 M NaCl Ni-NTA elution buffer as previously described for TelA. Peak fractions were identified by SDS-PAGE and pooled. For both SSB and SSBΔC7, the salt concentration of the combined Ni-NTA elutions was adjusted to 0.15 M NaCl with HG buffer and loaded to a 9 mL HS column. The HS columns were washed with 10 column volumes of 0.15 M NaCl HG followed by 1 column volume of 0.25 M NaCl HG and 1 column volume of 0.35 M NaCl HG. SSB and SSBΔC7 were eluted from the HS column with 18 mL of 0.5 M NaCl HG into 1.5 mL fractions. Peak fractions were identified by SDS-PAGE and tested for nuclease activity as described in the purification of TelA (107–442). Peak fractions lacking nuclease activity were pooled and the protein concentration determined with BioRad’s protein dye reagent [34].

### Oligonucleotide substrate annealing assay

All oligonucleotide annealing assays were performed in reaction buffer containing 25 mM HEPES (pH 7.6), 1 mM DTT, 2 mM CaCl_2_, 100 μg/mL BSA, and 50 mM potassium glutamate. For annealing reactions with ‘naked DNA’, 15 nM of 5’-^32^P endlabelled strand was combined with the reaction buffer and incubated on ice for 2 min, to inhibit spontaneous annealing, followed by addition of 15 nM of unlabelled strand and TelA with incubation at 30°C. For reactions with ‘SSB-complexed ssDNA’ or ‘SSBΔC7-complexed ssDNA’ both strands were separately preincubated with 105 nM of *Agrobacterium tumefaciencs* SSB (AtSSB) at 30°C for 5 min. The SSB-complexed oligonucleotides were then mixed and TelA was added to initiate the annealing reaction. Following the addition of TelA, all reactions were incubated at 30°C and 18 μL aliquots were removed from the 120 μL master reactions at 0.16, 0.5, 1, 2, and 4 min to monitor reaction progression. The 18 μL aliquots were mixed with pre-aliquoted SDS load dye containing an excess of an unlabelled version of the radiolabelled strand to prevent further annealing following termination of the reaction. For reactions pre-incubated with AtSSB or AtSSBΔC7, the SDS load dye was supplemented with 50 μg/mL pronase and the samples were deproteinated by incubation for a further 15 min at 30°C prior to gel loading. All annealing reactions were loaded to 8% PAGE 1XTAE/ 0.1%SDS gels and were electrophoresed at 13V/cm for 105 min. The gels were dried and exposed to phosphor-imaging screens for subsequent analysis on a phosphorimager. All reactions were performed in triplicate and data quantified with QuantityOne software. Reaction curves and statistics were generated with Prism’s GraphPad 6.0.

### Plasmid annealing assay

5’-^32^P endlabelled pUC19 ssDNA was prepared by digesting pUC19 DNA with BamHI followed by dephosphorylation with antarctic phosphatase, and by endlabelling with T4 polynucleotide kinase. Enzymes, ATP and buffer were removed/changed with G-25 Sephadex microspin columns. ssDNA was generated for the annealing experiments by heat treatment conducted in a PCR machine (99.9°C, 5 min) followed by snap cooling of the denatured DNA in ice water to prevent reannealing. Annealing reactions were performed in 25 mM HEPES (pH 7.6), 2 mM MgCl_2_, 1 mM DTT, 100 μg/mL BSA and 50 mM potassium glutamate. The annealing reactions contained 1.78 mM nucleotides of substrate DNA and 76 nM TelA and were incubated at 37°C for the times indicated. The samples were deproteinated by addition of SDS load dye to a 1X concentration and applied to an agarose gel. Gel electrophoresis was performed with a 0.7% agarose 1X TAE gel at 1V/cm for 15 h. The gels were dried onto P81 chromatography paper and exposed to a phosphor-imaging screen. The buffer conditions and protein concentrations are similar for SSB-complexed plasmid length annealing excepting use of CaCl_2_, no BSA, and 154 nM of TelA.

### Strand exchange assay

All strand exchange assays were performed in reaction buffer containing 25 mM HEPES (pH 7.6), 1 mM DTT, 2 mM CaCl_2_, 100 μg/mL BSA, and 50 mM potassium glutamate. TelA was preincubated at 30°C with 15 nM of either unlabelled donor DNA (OGCB411) or a 63 nt randomized sequence as a control (OGCB426). 15 nM of a 5’-^32^P radiolabelled partial duplex (OGCB410*/409) was then added to the reaction followed by further incubation at 30°C. 18 μL aliquots were removed from the master reaction at 0.16, 1, 2, 4 and 8 min timepoints and mixed with pre-aliquoted SDS stop dye containing an excess of cold OGCB410 to terminate the reaction. Strand exchange was monitored by displacement of the 410* oligonucleotide and results visualized on an 8% PAGE/ 1X TAE/ 0.1% SDS gel. The gels were dried and exposed to phosphor-imaging screens to be subsequently scanned by a phosphorimager.

## Results

### TelA promotes the annealing of complementary single-stranded DNA

**Fig 2:**
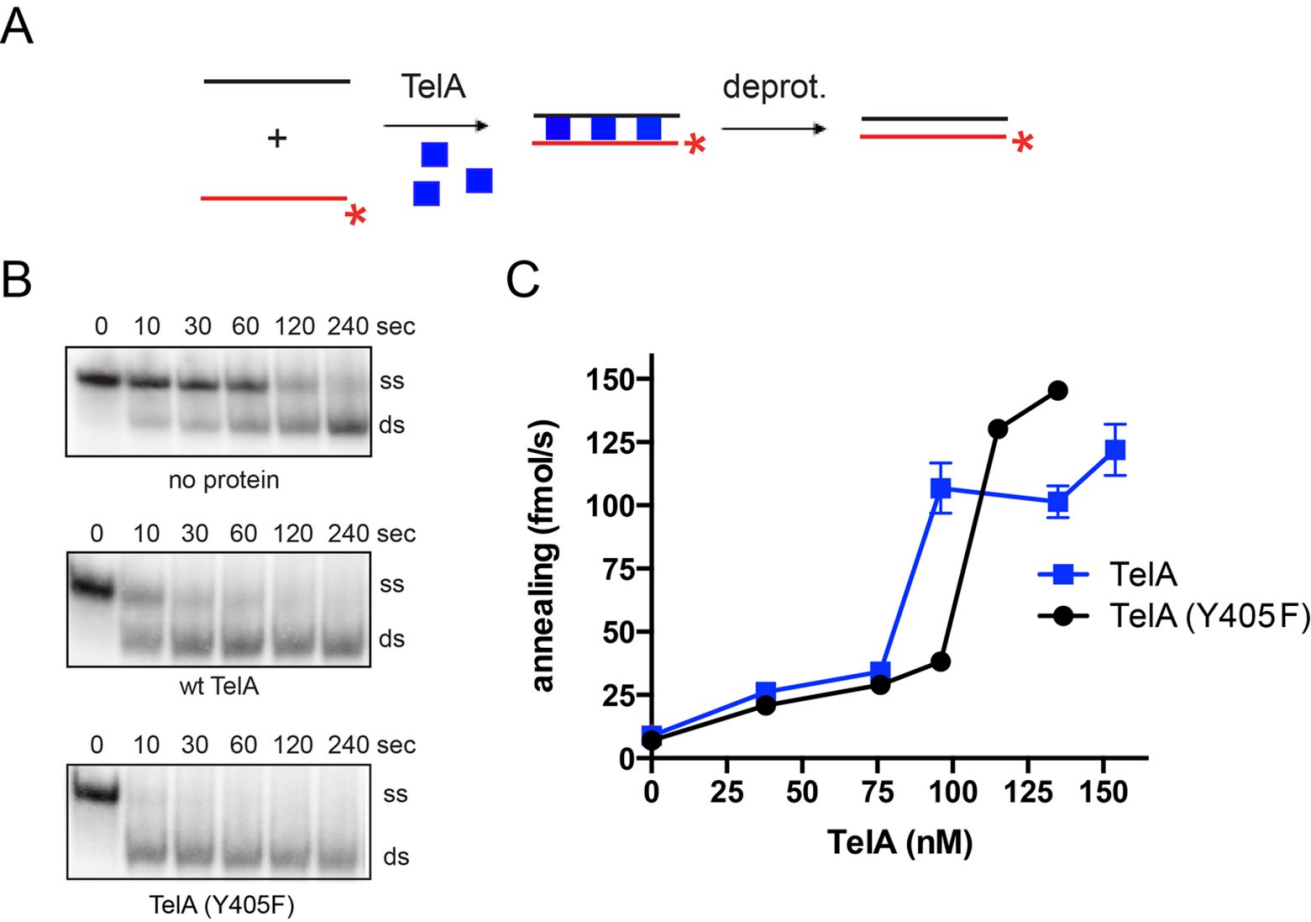
TelA is an annealing protein. A) A schematic representation of the singled-stranded annealing assay with naked DNA. The diagram shows the two complementary 87-nt oligonucleotides (OGCB455/456), with the 5’-^32^P endlabeled oligonucleotide represented in red and the unlabeled oligonucleotide shown in black. The 5’-^32^P endlabel is shown with the red asterisk. Addition of TelA to the reaction (blue squares) stimulates annealing of the two complementary oligonucleotides. B) Representative gel panels of 8%PAGE/1X TAE/0.1% SDS gel analysis for timecourse annealing reactions including spontaneous annealing (no protein), wildtype TelA, and the telomere resolution active site nucleophile mutant of TelA, TelA (Y405F). Where protein is present, gel panels using 135 nM of TelA are shown. The migration positions of the 5’-^32^P endlabeled reporter oligonucleotide (ss) and the duplex product (ds) are labeled. The electrophoretic mobility of native PAGE is affected by size, charge, and shape. The slower mobility of ss in this figure as compared to ds is likely due to the presence of secondary structure making ss less compact. C) A plot of annealing rate *vs*. TelA concentration is shown comparing wildtype TelA and the telomere resolution active site nucleophile mutant. The mean and standard deviation are shown and are derived from three independent experiments. Where error bars are not apparent the standard deviation was smaller than the points plotted.

Recent studies have shown that the borrelial telomere resolvase, ResT, in addition to its known telomere resolution activity, possesses a single-stranded annealing activity that extends to both naked DNA [27] and DNA complexed with its cognate SSB [28]. This suggests ResT is a multifunctional enzyme. While the agrobacterial telomere resolvase, TelA’s telomere resolution activity has also been demonstrated *in vitro* [20], there has yet to be any published studies on possible multifunctionality for TelA or any other telomere resolvase. We started with an *in vitro* characterization of TelA, testing the enzyme for annealing activity. For these assays, two complementary 87-nt oligonucleotides were used. One of the oligonucleotides was radiolabeled and used as the reporter strand while the other was left unlabeled. These oligonucleotides form an 83-nt duplexed product with 5’-GATC overhangs that can be visualized on a polyacrylamide gel as a change in the migration position of the reporter oligonucleotide (Fig 2A). Importantly, these reactions were terminated by SDS-load dye that denatures the enzyme and contains an excess of the unlabeled reporter oligonucleotide, essentially freezing the annealing reaction at the desired timepoint [35]. Protein free reactions, used to identify the spontaneous rate of annealing, were compared to those containing TelA. We found that TelA was capable of annealing DNA far above the spontaneous rate (Fig 2B), possessing a single-stranded DNA annealing activity similar to that of the borrelial telomere resolvase, ResT. An analysis of annealing rate *vs*. TelA concentration produced a non-linear response with a half-maximal rate between 70 and 100 nM of TelA (Fig 2C). TelA’s annealing activity was found to be less potent than ResT’s requiring a ~10-fold higher protein concentration to promote a half maximal annealing rate [27].

In addition to the wildtype enzyme, we also examined the telomere resolution active site nucleophile mutant of TelA, TelA (Y405F). This mutant is known to be inactive for telomere resolution [20] and was used to determine if a separation of function mutant defective only for telomere resolution could be identified. TelA (Y405F) did not eliminate annealing activity and displayed annealing capabilities similar to that of the wildtype enzyme (Fig 2B & 2C). The wildtype comparable annealing activity of TelA (Y405F) suggests distinct functional determinants, in TelA, for telomere resolution and single-stranded DNA annealing.

**Fig 3:**
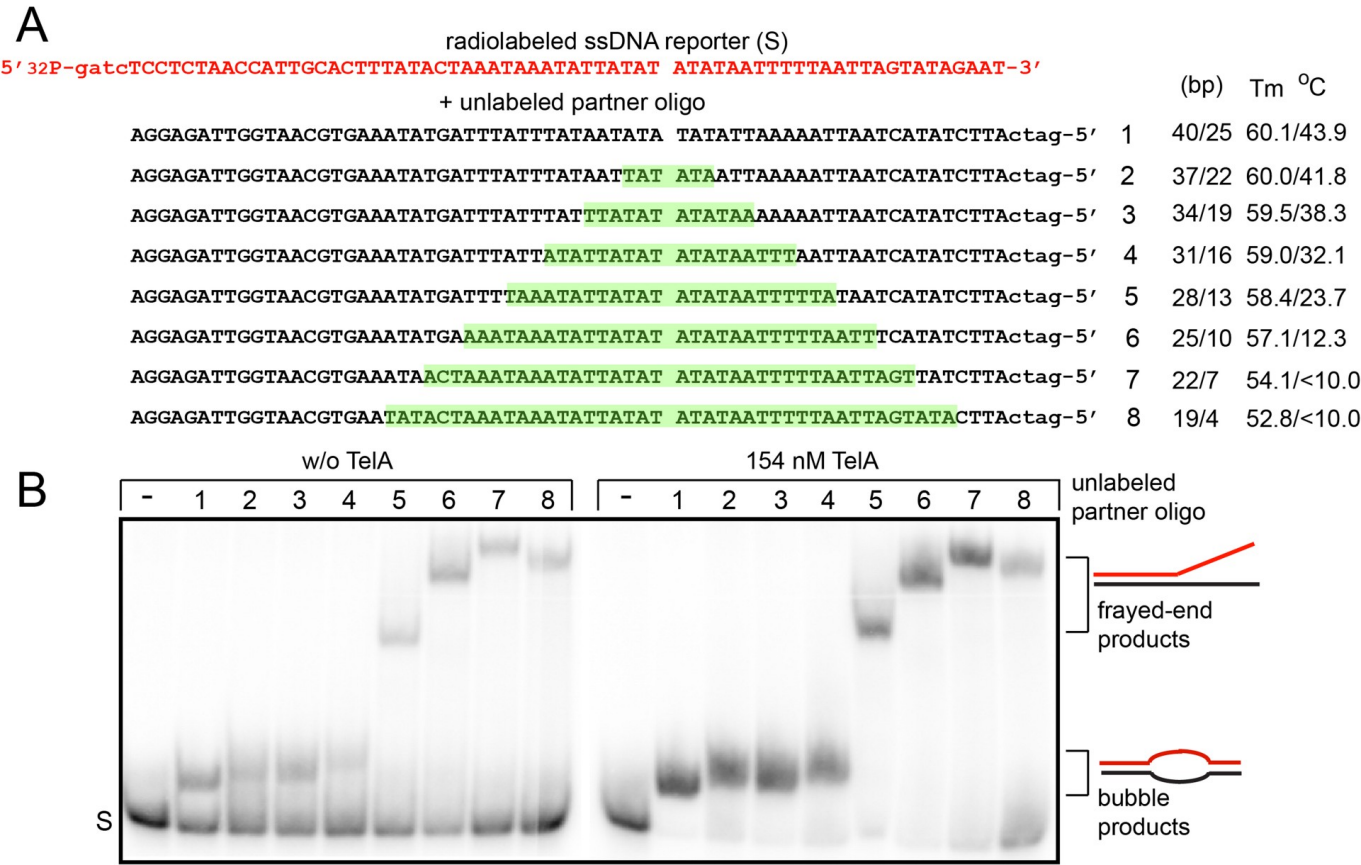
Determination of ssDNA length requirements for TelA-promoted single-stranded annealing. A) A schematic representation of the 69 nt 5’-^32^P endlabeled reporter oligonucleotide (red) and a set of eight unlabelled partner oligonucleotides (black) used in the single-stranded annealing assay. Oligonucleotide 1 is a fully complementary partner to the reporter oligo that anneals to form a 65-nt duplex with GATC 5’-overhangs. Each successive oligonucleotide introduces an additional 6-nt block of non-complementary sequence (highlighted in green), asymmetrically positioned to produce annealed products with different arm lengths and varying melting points. These are displayed to the right of the schematic. B) 8% PAGE/ 1X TAE/ 0.1% SDS gel panels of the spontaneous annealing (w/o TelA) and TelA-promoted annealing assays. The single-stranded annealing assays were performed as described in the Materials and Methods with each reaction being incubated for 20 s at 30°C prior to reaction termination and gel loading. S represents the migration position of the single-stranded reporter oligonucleotide. Bubble products are duplex products that are annealed at both ends despite lacking central base pairing, and frayed-end products are anchored at their left end only. The gel migration breakpoint between bubble products and frayed-end products was determined by observing the migration position of the reaction products in comparison to a modified version of oligo 5 that possesses only left flank homology and 41-nt of non-complementary sequence and thus, can only anneal into a fray-end product (see OGCB478 in S1 Table).

Next, we sought to determine the minimum ssDNA length requirements for TelA promoted single-stranded annealing and whether GC content affected these values. For these assays a radiolabelled reporter oligonucleotide was mixed with a variety of unlabelled partners that ranged from completely complementary partner strands to ones possessing progressively larger blocks of contiguous, internal non-complementary sequence (Fig 3A). The heterologous blocks of sequence were placed asymmetrically within the pairings to create left and right flanks of both differing sizes and GC content. Successful annealing of both flanks by TelA would yield a product possessing an unpaired bubble between the left and right flank. Conversely, an inability to successfully anneal the shorter, AT rich right flank would produce a frayed-end product. The migration breakpoint between bubble products and frayed-end products was determined empirically with a marker that’s right flank is entirely composed of heterologous sequence to intentionally produce a frayed-end product. TelA promoted single-strand annealing produces bubble products with reporter pairings possessing unpaired gaps of up to 18 nucleotides in length (Fig 3B). This corresponds to a minimum length requirement of 16 nucleotides of complementary sequence for the successful annealing of the AT rich right flank. This assay also reveals the GC rich left flank requires >19 nucleotides of complementary sequence as we observe reduced frayed-end product formation in the last oligonucleotide pairing.

### TelA can anneal ssDNA with strong secondary structure

**Fig 4:**
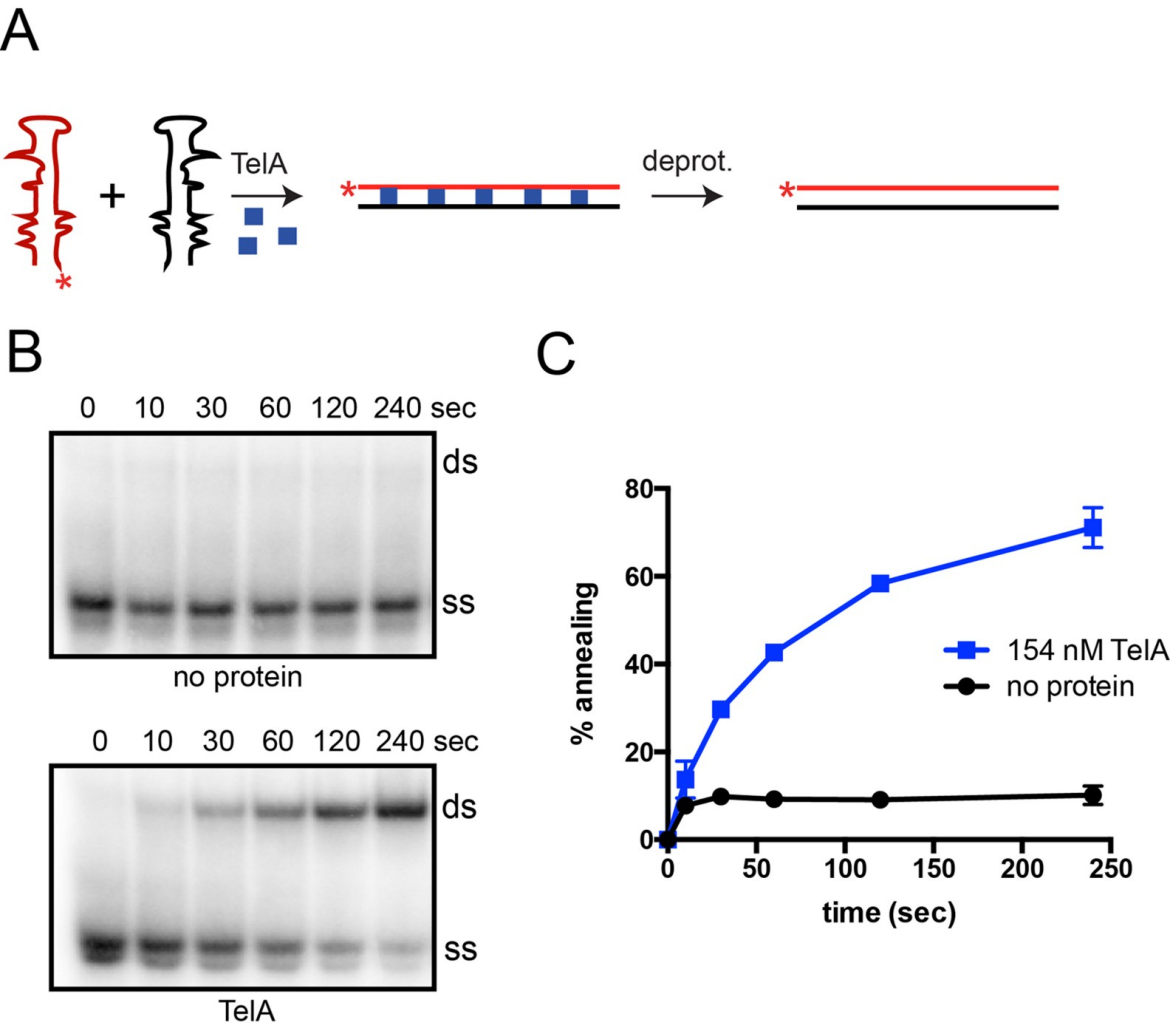
TelA anneals ssDNA possessing secondary structure. A) A schematic representation of the single-stranded annealing assay using substrates with complex secondary structure. These complementary DNA oligonucleotide sequences (red and black) are a mimic of an HIV transactivational response (TAR) element and naturally form a complex stem-loop structure with several bulges in their single-stranded form. Addition of TelA (blue squares) promotes removal of the secondary structure and subsequently stimulates annealing of the complementary strands into a lineform duplex. B) Representative 8% PAGE/ 1X TAE/ 0.1% SDS gel panels of timecourse annealing reactions with TAR sequences with or without TelA present. Where protein is present, reactions using 154 nM of TelA are shown. The migration positions of the 5’-^32^P endlabeled reporter TAR oligonucleotide (ss) and the duplex product (ds) are labeled. The electrophoretic mobility of native PAGE is affected by size, charge, and shape. Here ss migrates faster than ds, a contrast to Fig 2B. The TAR ss possesses significant internal base pairing making it extremely compact. C) A plot of annealing timecourses comparing spontaneous annealing and TelA-promoted annealing with TAR sequences. The mean and standard deviation are shown and are derived from three independent experiments.

To further categorize the nature of TelA’s annealing activity we examined TelA’s ability to anneal ssDNA possessing strong secondary structure. Single strand annealing assays were performed as previously described with the exception of the substrate oligonucleotides. We used a set of complementary DNA oligonucleotides that mimic the HIV transactivational response (TAR) element [36]. These substrates form a complex hairpin with multiple bulges in their single-stranded form and therefore, require TelA to remove the secondary structure present to promote successful single-stranded annealing (Fig 4A). We found that TelA was capable of annealing the TAR_DNA_ oligonucleotides (Fig 4B & 4C). The secondary structure present in the TAR oligonucleotides significantly reduced the spontaneous rate of annealing previously observed in our original annealing assays (Figs 2B & 4B).

**Fig 5:**
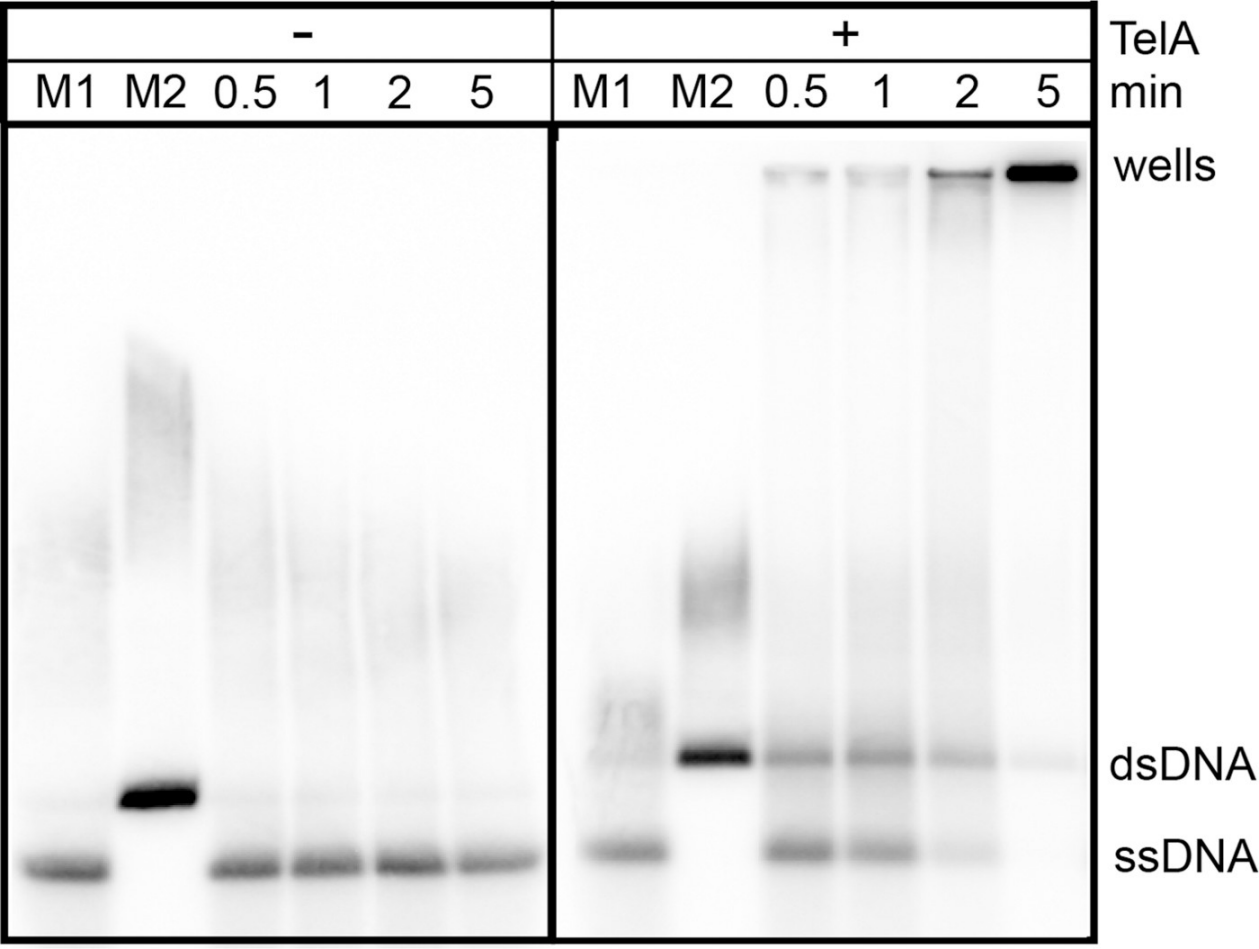
TelA can anneal plasmid length ssDNA. 0.7% agarose/ 1X TAE gel panels showing plasmid annealing reactions. The positions of heat denatured pUC19 (ssDNA), unit-length plasmid duplex (dsDNA) and the wells are labeled to the right of the gel. Reaction timepoints and the presence or absence of reaction components are indicated in the key above the gel. 76 nM of TelA was incubated with 1.78 mM nucleotides of the plasmid substrate in buffer containing 25 mM HEPES (pH 7.6), 2 mM MgCl_2_, 1 mM DTT, 100 μg/mL BSA and 50 mM potassium glutamate. pUC19 was linearized, ^32^P-endlabeled and denatured as described in the Materials and Methods section.

Having established that TelA could anneal ssDNA possessed of strong secondary structure we wanted to further establish whether TelA was capable of promoting long range annealing reactions. To investigate this, we tested whether TelA was capable of annealing plasmid length ssDNA. Across a variety of experimental conditions including a range of TelA concentrations and varying buffer conditions we observed two distinct reaction products. Under lower TelA concentrations and buffer conditions containing 2 mM MgCl_2_, the formation of a product representing the full annealed unit-length duplex can be observed (Fig 5) as well as a large amount of substrate residing in the wells of the gel that likely represents a network of partially annealed plasmid substrate. With alternative metal ions we observe more substrate accumulating in the wells and less unit-length dsDNA product (S1 Fig).

### TelA promotes limited strand exchange

**Fig 6:**
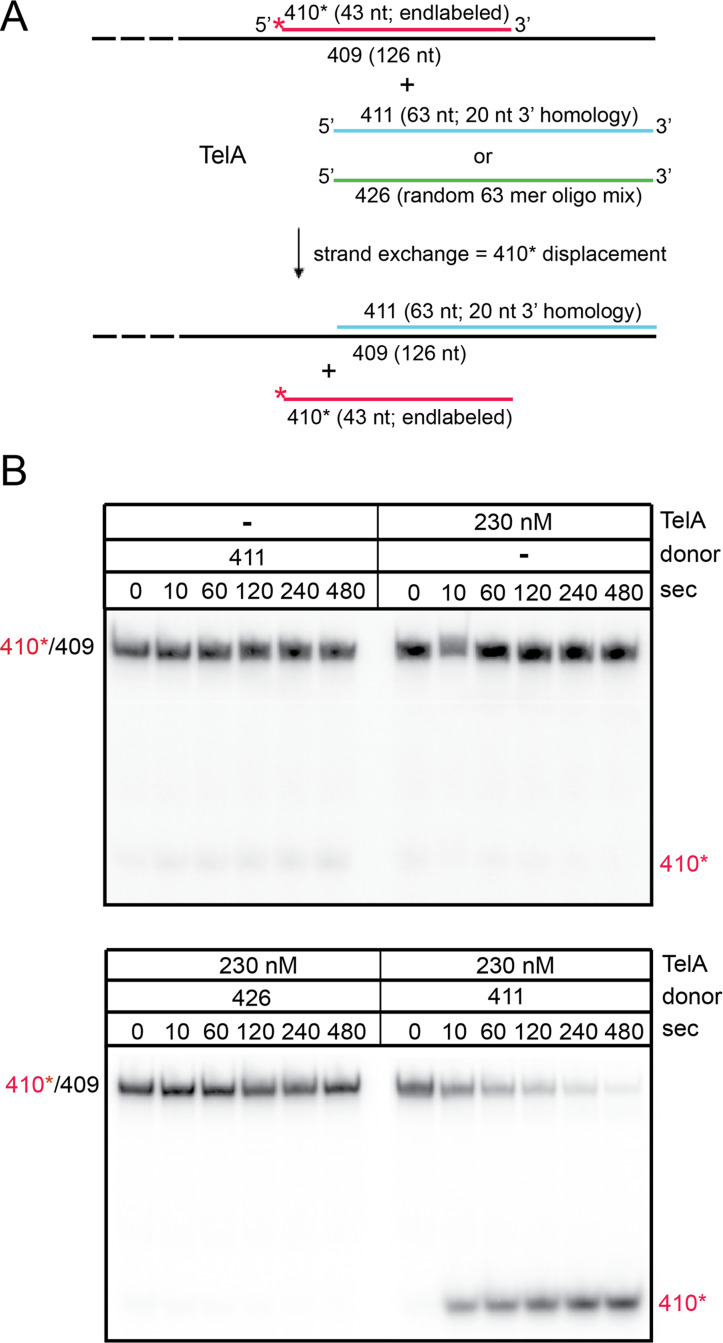
TelA promotes limited DNA strand exchange. A) A schematic representation of the DNA strand exchange and control reactions with a 5’-^32^P endlabeled 43-bp partially duplexed target (410*/409) and a 63-nt ssDNA homologous donor (411, blue) with 20 nt of 3’ flanking homology with the bottom strand of the partial duplex target or 63 nt of ssDNA of randomized sequence (426, green). The top strand of the partial duplex is red and the red asterisk represents the 5’ end label; the bottom strand is represented in black. B) 8% PAGE/ 1X TAE/ 0.1% SDS gel analysis of timecourse strand exchange reactions with combinations of TelA and various donors as indicated above the gels. The migration position of the partial duplex is labelled as 410*/409 and the migration of the displaced strand if strand exchange occurs is labelled as 410*.

The borrelial telomere resolvase, ResT, was previously shown to be able to perform limited strand exchange between a ssDNA donor and a homologous partial duplex DNA [27]. This activity is characteristic of other annealing proteins, such as phage λ‘s β protein, that can perform strand exchange via 3 strand branch migration [32]. We tested whether TelA was able to perform this kind of DNA strand exchange as well. This experiment involved preincubation of TelA with an unlabelled homologous donor strand followed by addition of a partial duplex target (radiolabelled on one strand) with further incubation at 30°C. The homologous donor strand is designed to anneal through 20 nt of complementarity to the 3’-ssDNA flank of the partial duplex target DNA, in principle, allowing the initiation of strand exchange via promotion of the annealing activity of TelA (Fig 6A).

Strand exchange was monitored by displacement of the radiolabelled strand of the duplex and visualized by differences in migration patterns on a polyacrylamide gel. The donor strand and the partial duplex were present in equimolar concentration in the reaction. We demonstrated here that TelA can perform limited strand exchange between a ssDNA donor strand and a homologous partial duplex substrate (Fig 6B). Displacement of the radiolabelled 410* strand only occurred in both the presence of TelA and of a homologous donor strand (OGCB411). Reaction with the non-homologous donor (OGCB426) did not result in strand exchange (Fig 6B).

### TelA promotes single-stranded annealing with SSB-complexed ssDNA

**Fig 7:**
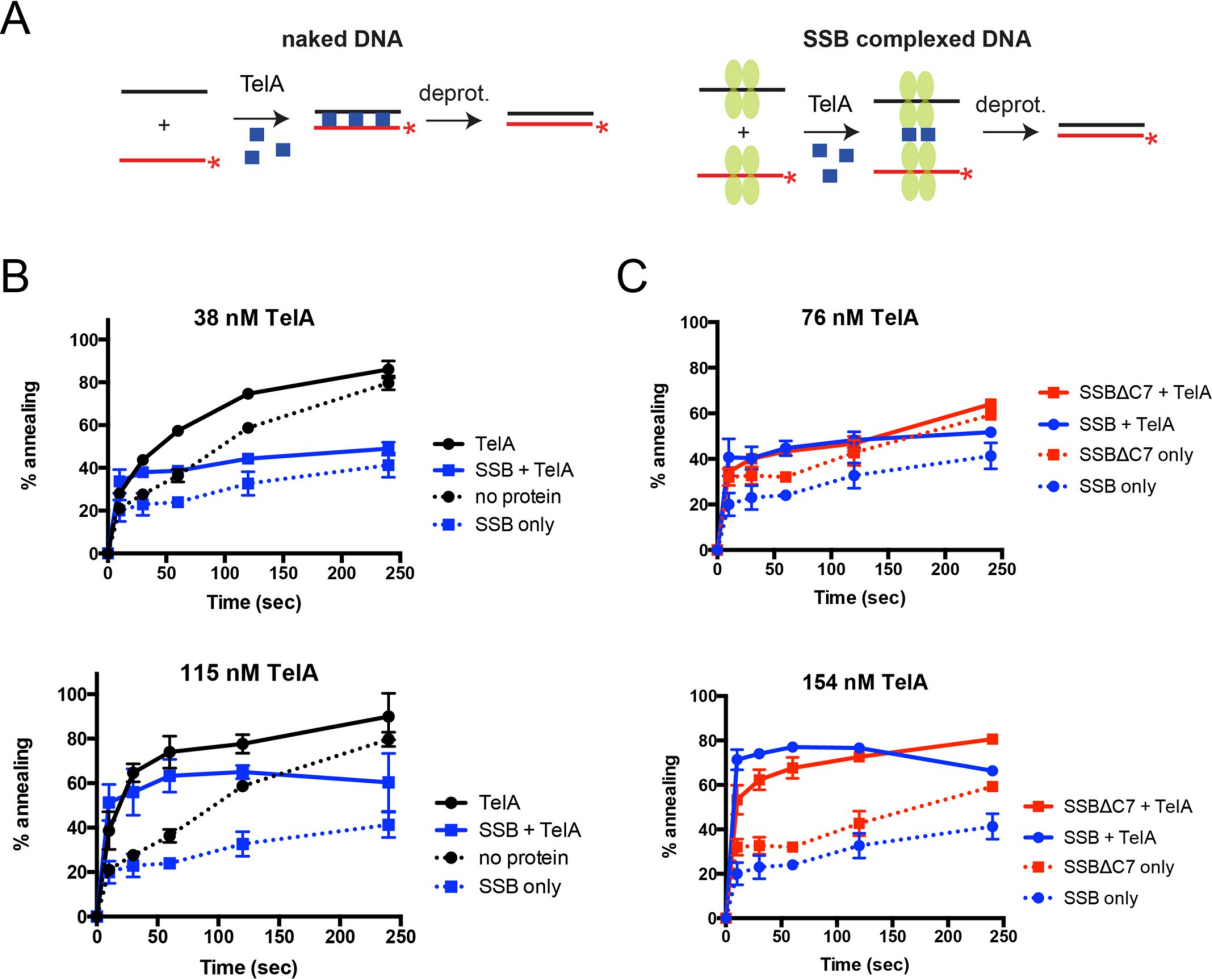
TelA promotes annealing with ssDNA complexed with its cognate single-stranded binding protein. A) A schematic representation of annealing assays with naked DNA and SSB-complexed DNA. Details of the experimental set up are as previously described in the legend to Fig 2 but with the addition of annealing reactions with SSB-complexed ssDNA. For annealing reactions with SSB-complexed DNA, the two complementary oligonucleotides are separately preincubated with SSB (green ovals). They are then mixed, followed by addition of TelA to initiate the annealing reaction. B) Plots of annealing timecourses comparing naked DNA and SSB-complexed DNA, with or without added TelA. Two plots are shown to represent timecourses performed with TelA concentrations below (38 nM) and above (115 nM) the equivalent molar concentration of added SSB (105 nM). The mean and standard deviation are shown and are derived from three independent experiments. C) Plots of annealing timecourses comparing TelA with SSB-complexed DNA (blue) and SSBΔC7 complexed DNA (red), with or without TelA. Two plots are shown to represent timecourses performed with TelA concentrations below (76 nM) and above (154 nM) the equivalent molar concentration of added SSB (105 nM). The mean and standard deviation are shown and are derived from three independent experiments.

ResT’s single-stranded DNA annealing activity was previously shown to extend to ssDNA complexed with its cognate SSB and to operate via specific ResT-SSB interaction mediated by SSB’s conserved amphipathic C-terminal tail [28]. We wanted to test whether TelA possessed this additional ability as well. Annealing assays similar to those previously described for reactions with naked ssDNA were used. Additionally, both complementary oligonucleotides were separately preincubated with 105 nM of AtSSB and then combined prior to the addition of TelA (Fig 7A). Coating the ssDNA with SSB appeared to suppress spontaneous annealing. At low concentrations of TelA we do not observe significant annealing activity. When TelA was present at a molar equivalent amount with SSB or greater (≥105 nM) we observed significant TelA-promoted ssDNA annealing activity (Fig 7B).

SSB’s are ubiquitous in nature and research on *E*. *coli*’s (Ec) SSB has identified interactions with DNA replication, recombination, repair, and replication restart functions; all of which are mediated through protein-protein interactions with EcSSB’s highly conserved amphipathic C-terminal tail consisting of an acidic triad (DDD) and final hydrophobic residues [37]. Both the borrelial and agrobacterial SSB are identifiable homologues of EcSSB and possess this conserved C-terminal tail (S2 Fig). We truncated the last seven amino acids of AtSSB that encompass these conserved residues to produce AtSSBΔC7. AtSSB and AtSSBΔC7 were compared to one another using electrophoretic mobility shift assays and protein-protein crosslinking to determine if deletion of the C-terminal tail produced any observable effects on ssDNA binding affinity or SSB oligomerization status. There was no observable difference between the wildtype AtSSB and the C-terminal truncation of SSB’s oligomerization properties; SSBΔC7 did display a slightly reduced affinity for ssDNA in comparison to wildtype SSB (S3 Fig). While ResT was unable to support annealing of ssDNA complexed with its truncated SSB [28], TelA displays an annealing activity on AtSSBΔC7-coated ssDNA comparable to that of wildtype AtSSB (Fig 7C). This suggests that an interaction with the C-terminal tail of SSB is not essential to TelA promoted single-stranded annealing of SSB-coated DNA.

**Fig 8:**
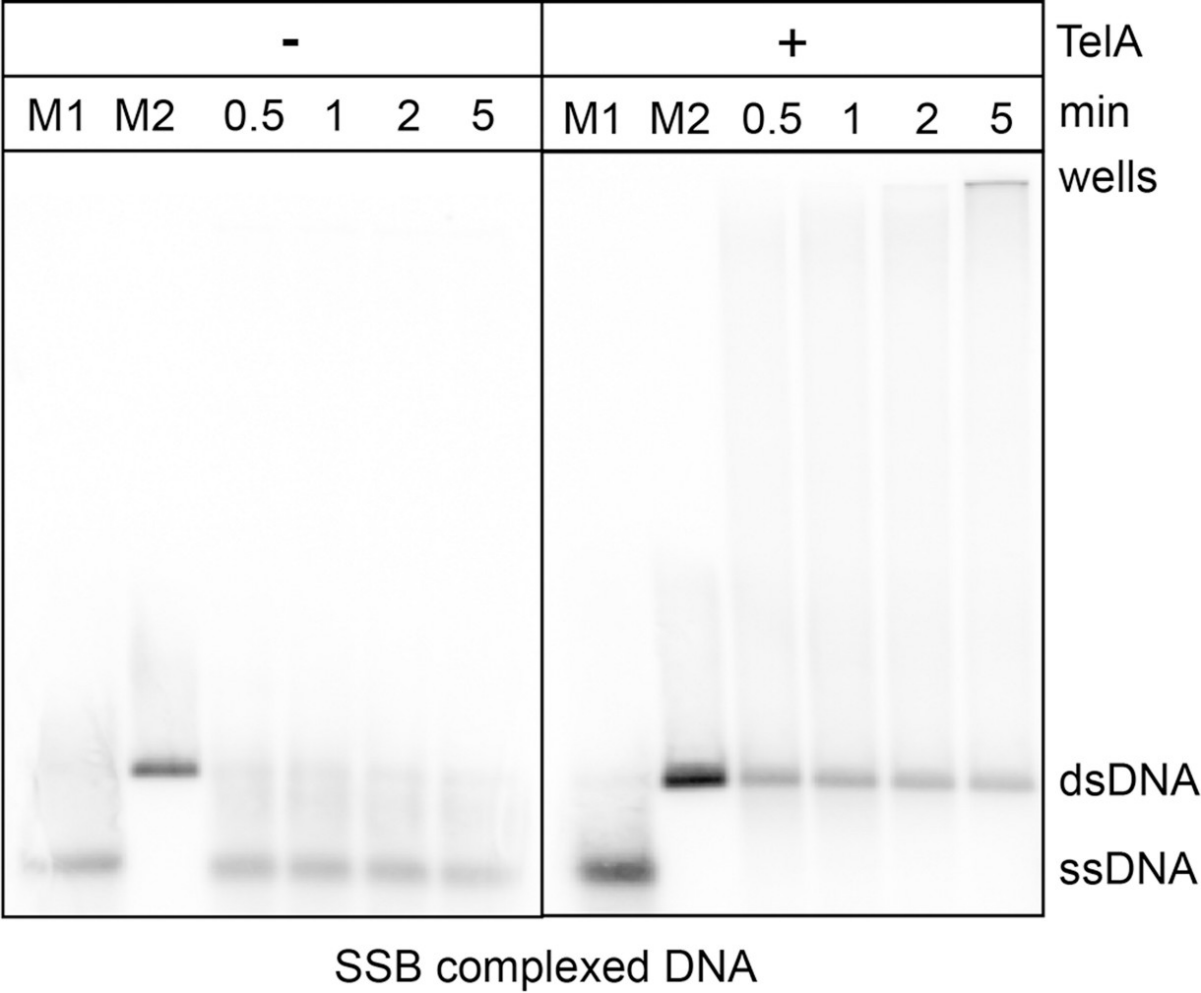
TelA promotes annealing of plasmid length DNA complexed with its cognate SSB. 0.7% agarose/ 1X TAE gel panels showing SSB-complexed plasmid annealing reactions. The migration patterns of heat denatured pUC19 (ss) and the unit-length plasmid duplex (ds) are labeled as shown. Reaction timepoints and the presence or absence of reaction components are indicated in the key above the gel. 1.78 mM nucleotides of heat denatured pUC19 was preincubated with 105 nM of SSB in buffer containing 25 mM HEPES (pH 7.6), 1 mM DTT, 2 mM CaCl_2_, and 50 mM potassium glutamate. Following pre-incubation with SSB, annealing was initiated by the addition of 154 nM of TelA.

TelA is also capable of annealing SSB-complexed plasmid length DNA. Reactions with naked plasmid length DNA were sensitive to high TelA concentrations and varying buffer conditions with most of the observable product accumulating in the wells (S1 Fig). With 154 nM of TelA we observed SSB-complexed plasmid length ssDNA annealed into a unit-length dsDNA product with almost no partially annealed substrate accumulating in the wells (Fig 8). SSB appears to reduce the complex secondary structure present in plasmid length DNA thereby facilitating long range annealing.

### Deletion of the N-terminal domain of TelA abolishes its annealing activity

**Fig 9:**
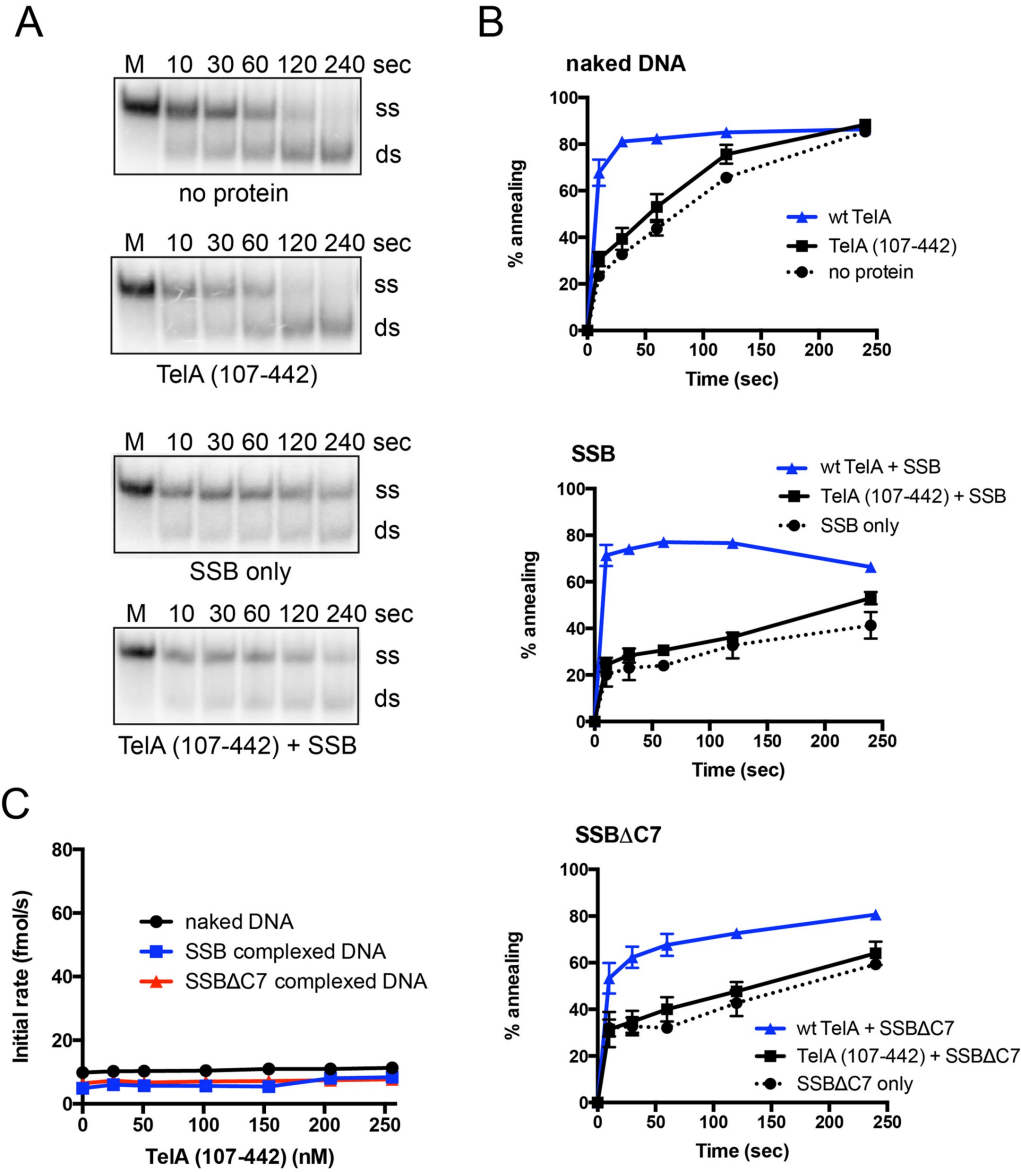
Deletion of the N-terminal domain of TelA abolishes TelA’s annealing activity. A) Representative 8% PAGE/ 1X TAE/ 0.1% SDS gel panels of annealing assay timecourses comparing reactions of naked ssDNA with and without 154 nM TelA (107–442), and SSB-complexed ssDNA with and without 154 nM TelA (107–442). The migration positions of the 5’-^32^P endlabeled reporter oligonucleotide (ss) and the duplexed product (ds) are indicated. B) Plots of annealing timecourses with naked DNA, SSB-complexed DNA, or SSBΔC7 complexed DNA as labeled. Each plot compares the spontaneous rate of annealing, TelA (107–442) promoted annealing, and wildtype TelA promoted annealing under the indicated conditions. All reactions shown were performed with 154 nM of TelA, and 105 nM of SSB or SSBΔC7 where appropriate. C) A plot of annealing rate *vs*. TelA (107–442) concentration is shown comparing annealing with naked DNA, SSB-complexed DNA, and SSBΔC7 complexed DNA. The mean and standard deviation are shown and are derived from three independent experiments.

It was previously reported that the N-terminal domain of TelA was dispensable for telomere resolution [20]. Additionally, a set of telomere DNA-TelA cocrystal structures showed that the TelA structure only began to resolve at residue 102, suggesting the N-terminal domain of TelA may have alternative functions [16]. We constructed an N-terminal truncation mutant of TelA to assess whether removal of its N-terminal domain had an effect on annealing activity. TelA (107–442) was active as a telomere resolvase (S4 Fig. & [20]) but was unable to anneal complementary ssDNA above the spontaneous rate (Fig 9). This was true across a wide TelA (107–442) concentration range (Fig 9C). Additionally, the annealing activity could not be rescued by replacing naked ssDNA with either SSB-complexed or SSBΔC7-complexed ssDNA (Fig 9B & 9C). These data suggest the N-terminal domain of TelA to be essential for the annealing activity. While a similar N-terminal truncation mutant has been previously characterized in ResT, ResT (164–449), this mutant does not retain either its ssDNA annealing or telomere resolution activity [17, 27, 28]. Thus, ResT (164–449) does not possess the same separation of function as TelA (107–442).

### The N-terminal domain of TelA possesses annealing activity

**Fig 10:**
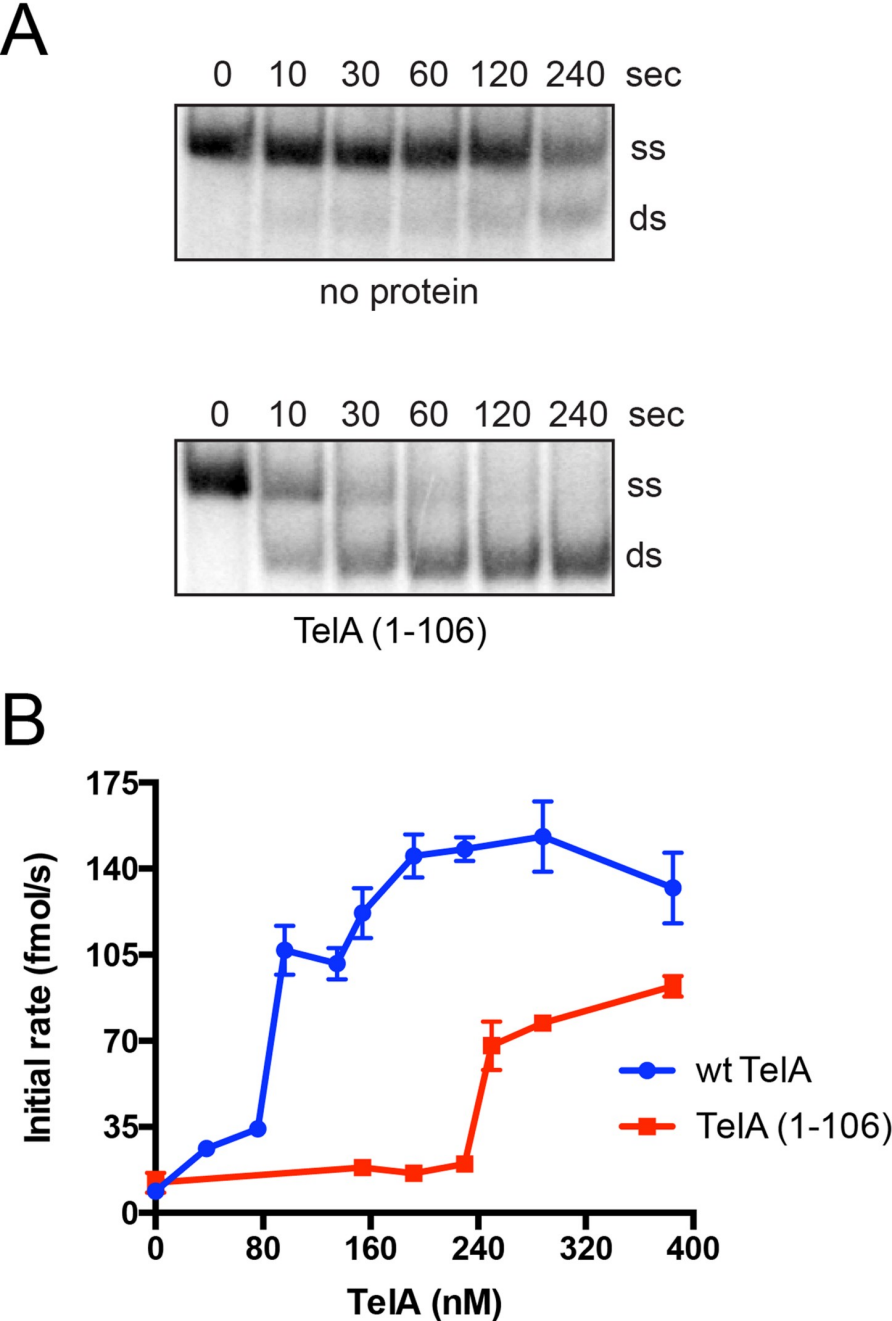
TelA (1–106) is an annealing protein. A) Representative 8%PAGE/ 1X TAE/ 0.1% SDS gel panels of annealing timecourses comparing spontaneous annealing (no protein) to reactions containing 288 nM of the N-terminal domain of TelA (TelA (1–106)). The migration positions of the 5’-^32^P endlabeled reporter oligonucleotide (ss) and the duplex product (ds) are indicated. B) A plot of annealing rate vs. TelA concentration is shown comparing the annealing rates of wildtype TelA (blue) and TelA (1–106) (red). The wildtype TelA data ranging from the spontaneous rate of annealing to 154 nM of TelA was previously shown in Fig 2C. Data for annealing rates with higher concentrations of wt TelA (192 nM– 385 nM) were collected in tandem with TelA (1–106) annealing rates and have not been previously shown. The mean and standard deviation are shown and are derived from three independent experiments.

The inability of the N-terminal truncation mutant of TelA to anneal ssDNA above the spontaneous rate suggests TelA’s N-terminal domain to be essential for its annealing activity. We independently expressed and purified TelA’s N-terminal domain, TelA (1–106), to assess the domain for potential annealing activity. The N-terminal domain of TelA possessed ssDNA annealing activity (Fig 10A), though requiring higher protein concentrations than the full-length protein to observe robust single-stranded annealing (Fig 10B). TelA (1–106) was also active as an annealing protein on SSB-complexed ssDNA and ssDNA possessing strong secondary structure (S5 & S6 Figs). In addition to its annealing activity TelA (1–106) was also able to perform limited strand exchange; similar to the annealing reactions, strand exchange required higher TelA (1–106) concentrations than reactions conducted with wildtype TelA (S7 Fig). Collectively, these data suggest TelA’s annealing activity stems from its N-terminal domain and further solidifies a separation of function between TelA’s N-terminal ssDNA annealing and C-terminal residing telomere resolution activities.

## Discussion

We have demonstrated here that the agrobacterial telomere resolvase, TelA, possesses singled-stranded DNA annealing activity in addition to its previously reported telomere resolution activity (Fig 2 & [20]). This activity extended to ssDNA possessing complex secondary structure, plasmid length ssDNA (Figs 4 & 5) and ssDNA complexed with its cognate SSB (Fig 7B). Furthermore, we demonstrated that TelA’s single-strand annealing activity can promote a limited strand exchange reaction typical of those promoted by annealing recombinases like λ phage’s β protein (Fig 6 and S7 Fig).

The borrelial telomere resolvase, ResT, was previously shown to anneal ssDNA, to anneal SSB-complexed ssDNA and to promote limited strand exchange reactions [27, 28]. Characterization of the ResT-promoted annealing of ssDNA bound by its cognate SSB implied a specific physical and functional interaction between ResT and the conserved C-terminal tail of SSB, as ResT could not promote the annealing of ssDNA complexed with a C-terminal truncation of SSB, BbSSBΔC5. This specific ResT-SSB interaction was confirmed by multiple physical interaction assays [28]. Conversely, in this report we have shown that TelA was still able to anneal ssDNA complexed with a similar C-terminal truncation mutant of the agrobacterial SSB, AtSSBΔC7 (Fig 7C) despite AtSSB displaying strong homology with the conserved C-terminal tails of EcSSB and BbSSB (S2 Fig). This suggested that either TelA does not interact directly with its cognate SSB or does not do so through the conserved C-terminal tail. Consistent with the view that a specific TelA-SSB interaction may not be necessary to promote annealing was our inability to detect TelA-SSB interactions using TelA-SSB crosslinking and TelA-SSB coshift assays on ssDNA that were used to detect specific ResT-SSB interactions in the previous report of ResT-SSB/ssDNA annealing (S8 and S9 Figs and [28]). In the mycobacterial RecOR system, RecO possesses a similar ability to anneal complementary SSB-complexed ssDNA independent of the usual expected SSB-Ct interaction [38]. It is presently unknown if TelA-promoted annealing of SSB-bound ssDNA proceeds via a ternary complex, as was shown for ResT [28], or involves a replacement of SSB with TelA. It remains possible a ternary complex that is not stable enough to be detected by the co-shift and crosslinking studies performed, to date, is involved.

We have further shown that TelA’s annealing activity stems primarily from its N-terminal domain as deletion of the N-terminal domain abolished TelA’s annealing activity (Fig 9) and the N-terminal domain of TelA functioned independently in annealing and strand exchange assays (Fig 10 and S7 Fig). For ResT the N-terminal domain was only able to anneal naked ssDNA; important ResT-SSB interactions had been lost by deletion of the rest of the protein as evidenced by ResT (1–163)’s inability to anneal ssDNA bound by SSB [28]. In contrast, the N-terminal domain of TelA expressed as an independent protein was able to anneal both naked ssDNA and ssDNA bound by its cognate SSB, consistent with a lack of specific TelA-SSB interactions needed to promote annealing of SSB bound ssDNAs (Fig 10 and S6 Fig).

In addition to the N-terminal deletion mutant that eliminated the annealing activity without affecting telomere resolution, we have shown that a mutant of the active site nucleophilic tyrosine of the telomere resolvase domain, TelA (Y405F), displayed wildtype comparable levels of annealing activity (Fig 2). Collectively, these mutants represent a clean separation of function between TelA’s telomere resolution activity and the newly identified annealing activity reported here. A larger N-terminal truncation mutant of the borrelial telomere resolvase, ResT (ResT (163–449)) has been previously reported, and although removal of the N-terminal domain similarly abolished the ResT’s annealing activity it also abrogated telomere resolution [17].

Single strand annealing and strand exchange activity have now been demonstrated for two telomere resolvases that are implicated in completing chromosome replication in *Borrelia burgdorferi* and *Agrobacterium tumefaciens*, implying that the annealing activity serves some important, conserved, function *in vivo*. For the borrelial telomere resolvase, ResT, we speculated that the annealing activity, operative even on SSB-coated ssDNA may be a functional analogue of RecOR functions in *B*. *burgdorferi*, since this bacterium lacks identifiable RecOR homologues. However, our discovery that TelA also possesses these activities casts doubt on that hypothesis, since the agrobacterial genome does possess RecOR homologues (Atu1039 and Atu0093 in KEGG database of *Agrobacterium tumefaciens* genome; https://www.genome.jp/kegg-bin/show_organism?org=atu). It is possible that TelA confers a redundant activity for RecOR but it seems increasingly likely that the annealing activity noted for two telomere resolvases in distantly related bacterial species indicates a conserved function in hairpin telomere metabolism. In any case, the N-terminal truncation mutant of TelA presented here along with TelA (Y405F) provide a platform for future *in vivo* functional studies aimed at testing these hypotheses and identifying the role of the annealing activity in the cell.

## Supporting information

S1 Fig Effect of metal ions on TelA’s ability to anneal plasmid length DNA. 0.7% agarose/ 1X TAE gel panel showing plasmid annealing reactions under various metal conditions. The migration patterns of heat denatured pUC19 (ssDNA) and the unit-length plasmid duplex (dsDNA) are labeled as shown. Reaction timepoints are indicated in the key above the gel. 76 nM of TelA was incubated with 1.78 mM nucleotides of the plasmid substrate in buffer containing 25 mM HEPES (pH 7.6), 1 mM DTT, 100 μg/mL BSA and 50 mM potassium glutamate. Reactions contained either 1 mM EDTA, 2 mM MgCl_2_, or 2 mM CaCl_2_ as labeled on the gel. The pUC19 was linearized and ^32^P-endlabeled as described in the Materials and Methods section.
(TIF)

S2 Fig AtSSB comparison with *E. coli* and *B. burgdorferi* SSB. A) A multiple sequence alignment comparing SSB proteins from *Escherichia coli*, *Agrobacterium tumefacien*s, and *Borrelia burgdorferi*. Accession numbers: P0AGE0, SSB from *Escherichia coli*; O51141, SSB from *Borrelia burgdorferi*; Q8UF87, SSB from *Agrobacterium tumefaciens*. Identical amino acids are denoted by asterisks (*) and similar side chains by two dots (:). B) The conserved C-terminal tail identified in *Escherichia coli* (EcSSB) and *Borrelia burgdorferi* (BbSSB) SSBs is also present in *Agrobacterium tumefaciens* SSB (AtSSB). They share 100% similarity with one another in respect to their last seven amino acids. AtSSB shares stronger identity with EcSSB (85.7%) than BbSSB does at these positions.
(TIF)

S3 Fig SSBΔC7 exhibits a slightly reduced ssDNA binding affinity in comparison to wildtype SSB and similar multimerization properties. A) 8% PAGE/ 1X TAE gel of an electrophoretic mobility shift assay of wildtype AtSSB or AtSSBΔC7 with a 35-nt ssDNA reporter, OGCB664. Binding reactions contained 15 nM of 5’-^32^P endlabeled OGCB664 in 25 mM HEPES (pH 7.6), 0.1 mM EDTA and 50 mM potassium glutamate with variant concentrations of SSB as indicated in the key above the gel. Binding reactions were performed at 20°C for 10 min prior to reaction termination with loading dye containing no SDS to a 1X final concentration. B) Coomassie stained 5%/13% SDS-PAGE gel of AtSSB protein-protein crosslinking with or without unlabeled OGCB664 ssDNA. These reactions were used to assess the oligomeric status of AtSSB in solution and bound to ssDNA. 7.8 μM of AtSSB was incubated with or without 975 nM nucleotides of OGCB664 in 25 mM HEPES (pH 7.6), 0.1 mM EDTA and 160 mM potassium glutamate for 10 min at 20°C. Crosslinking was induced by addition of glutaraldehyde to final concentrations as indicated in the key above the gel followed by continued incubation at 20°C for 5 min. Excess crosslinker was quenched with Tris (pH 8.5) to a final concentration of 100 mM and additional incubation at 20°C for 5 min prior to gel loading. C) Coomassie stained 5%/13% SDS-PAGE gel of AtSSBΔC7 protein-protein crosslinking with or without unlabeled OGCB664 ssDNA. These reactions were used to assess the oligomeric status of AtSSBΔC7 in solution and bound to ssDNA. Reaction conditions are as reported in B).
(TIF)

S4 Fig TelA (107–442) is active as a telomere resolvase. A) Schematic of the telomere resolution assay with a plasmid substrate. A pUC19 plasmid containing a 36 bp *rTel* sequence (grey, shaded area) was linearized by SspI. 1.75 μg/mL of the linearized plasmid (S) was combined with buffer containing 25 mM HEPES (pH 7.6), 1 mM DTT, 1 mM CaCl_2_, 100 μg/mL BSA, and 50 mM potassium glutamate. 19 nM of either wt TelA or TelA (107–442) was added to the reactions and incubated at 30°C. The conversion of (S) into two hairpin products (P1 and P2) was monitored by the removal of aliquots of the reaction mixture at indicated timepoints and combining them with SDS loading dye to 1X final concentration. B) Ethidium bromide stained 0.8% agarose/ 1X TAE gel panels showing telomere resolution timecourses with 19 nM of wt TelA and TelA (107–442).
(TIF)

S5 Fig TelA (1–106) can anneal ssDNA with complex secondary structure. A plot of annealing timecourses comparing spontaneous annealing and annealing with 385 nM of TelA (1–106) with TAR substrates. The mean and standard deviation are shown and are derived from three separate experiments.
(TIF)

S6 Fig TelA (1–106) can anneal ssDNA complexed with its cognate SSB. Plots of annealing timecourses with SSB-complexed DNA comparing reactions with and without 385 nM of TelA (1–106). SSB was present at 105 nM.
(TIF)

S7 Fig TelA (1–106) promotes limited strand exchange. Representative 8% PAGE/ 1X TAE/ 0.1% SDS gel analysis of timecourse strand exchange reactions with and without TelA (1–106) present (385 nM). The migration position of the partial duplex is labelled as 410*/409 and the migration of the displaced strand if strand exchange occurs is labelled as 410*.
(TIF)

S8 Fig TelA does not coshift with AtSSB or AtSSBΔC7 bound ssDNA. 7% PAGE/ 0.5X TBE gel of an electrophoretic mobility (co)shift assay of TelA with either AtSSB or AtSSBΔC7 and a 5’ radiolabeled 35-nt oligonucleotide (664*). AtSSB or AtSSBΔC7 was preincubated at 20°C for 10 min with 15 nM 664* in buffer containing 25 mM HEPES (pH 7.6), 1 mM DTT, 2 mM CaCl_2_, and 40 mM potassium glutamate. TelA was then added in a range of concentrations, as indicated in the legend, and the salt concentration adjusted to 100 mM potassium glutamate. The reactions were then further incubated for an additional 10 min prior to the addition of load dye containing no SDS.
(TIF)

S9 Fig Protein-protein crosslinking with glutaraldehyde cannot capture a TelA-SSB interaction. A) Protein-protein crosslinking performed in buffer containing 25 mM HEPES (pH 7.6), 0.1 mM EDTA, and 100 mM NaCl. TelA, AtSSB, and AtSSBΔC7 were present as indicated in the legend. Reactions were incubated at 30°C for 15 min. Crosslinking was induced by glutaraldehyde to a final concentration of 0.0001% followed by further incubation for 5 min. Excess crosslinker was quenched with Tris (pH 8.5) to a final concentration of 100 mM and additional incubation at 20°C for 5 min prior to gel loading. The results were visualized by application to a 3.5%/ 4–15% SDS-PAGE gradient gel followed by western blotting with a monoclonal antibody that recognizes the N-terminal 6X His tag on TelA and the SSBs. B) Protein-protein crosslinking performed in buffer containing 200 mM NaCl. Otherwise as described in A).
(TIF)

S1 Table Oligonucleotides used for substrate and mutant plasmid construction. For all listed primers, the codon highlighted in red indicates the amino acid being altered from the parental strand.
(DOCX)

S1 Sequence Synthetic TelA gene sequence. The gene sequence and corresponding amino acid sequence for the synthetic TelA gene are shown and numbered, respectively. The NdeI and BamHI restriction sites are **bolded** and the stop codon introduced into the synthetic gene is highlighted in red. The synthetic gene was blunt-end cloned into pUCIDT by IDT and verified by DNA sequencing. This figure was generated from https://www.bioinformatics.nl/cgi-bin/emboss/prettyseq.
(DOCX)

S2 Sequence Synthetic *Agrobacterium* SSB gene sequence. The gene sequence and corresponding amino acid sequence for the synthetic AtSSB gene are shown and numbered, respectively. The NdeI and BamHI restriction sites are **bolded** and the stop codon introduced into the synthetic gene is highlighted in red. The synthetic gene was blunt-end cloned into pUCIDT by IDT and verified by DNA sequencing. This figure was generated from https://www.bioinformatics.nl/cgi-bin/emboss/prettyseq.
(DOCX)

S1 Raw images (PDF)
