# Supplementary figures and images for: Single stranded DNA annealing is a conserved activity of telomere resolvases
Source: PLoS One. 2021 Feb 4;16(2):e0246212. doi: 10.1371/journal.pone.0246212 (PMC7861564; doi:10.1371/journal.pone.0246212)

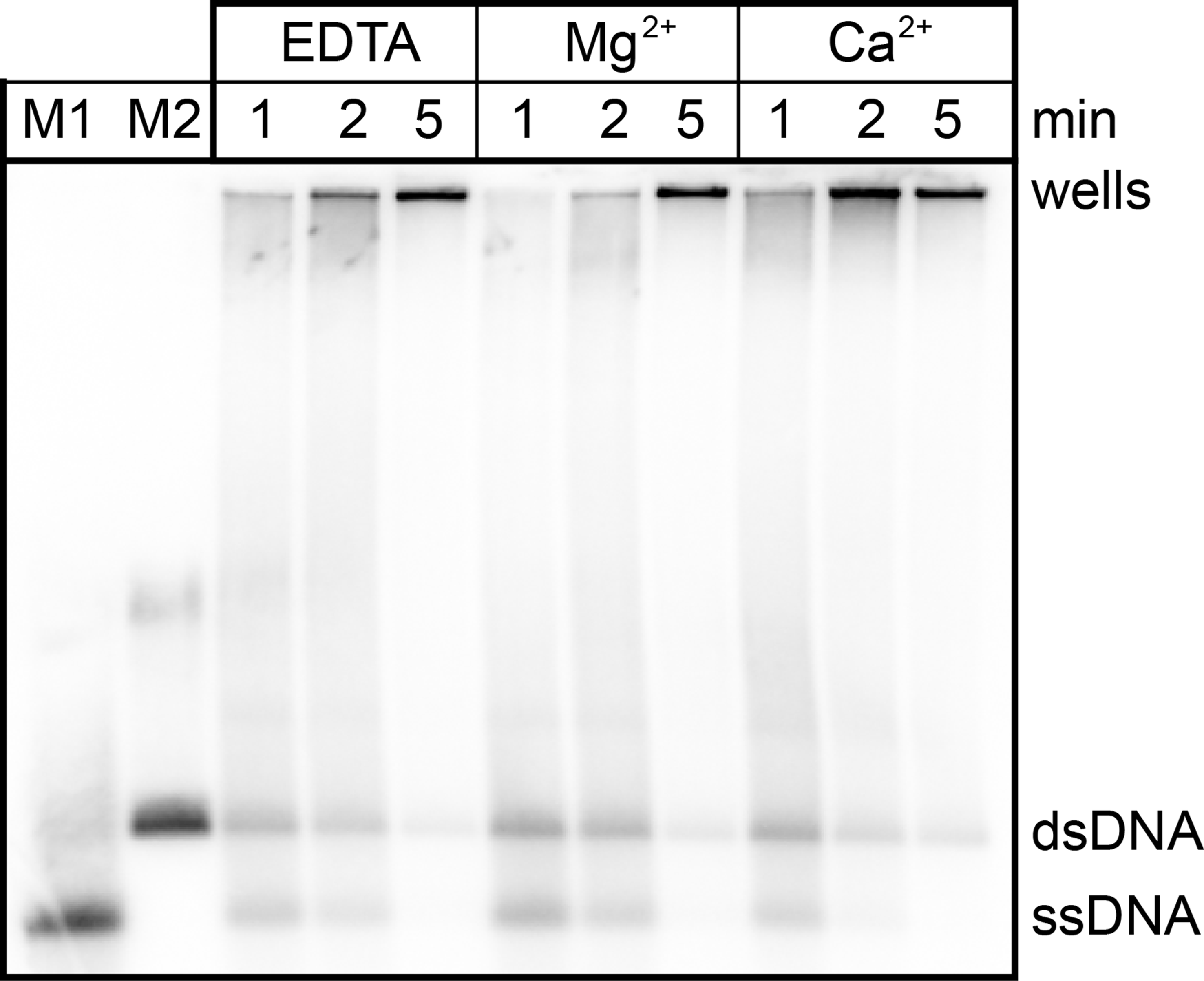

Supplement: S1 Fig — 0.7% agarose/ 1X TAE gel panel showing plasmid annealing reactions under various metal conditions. The migration patterns of heat denatured pUC19 (ssDNA) and the unit-length plasmid duplex (dsDNA) are labeled as shown. Reaction timepoints are indicated in the key above the gel. 76 nM of TelA was incubated with 1.78 mM nucleotides of the plasmid substrate in buffer containing 25 mM HEPES (pH 7.6), 1 mM DTT, 100 μg/mL BSA and 50 mM potassium glutamate. Reactions contained either 1 mM EDTA, 2 mM MgCl2, or 2 mM CaCl2 as labeled on the gel. The pUC19 was linearized and 32P-endlabeled as described in the Materials and Methods section. (TIF) [file pone.0246212.s001.tif]

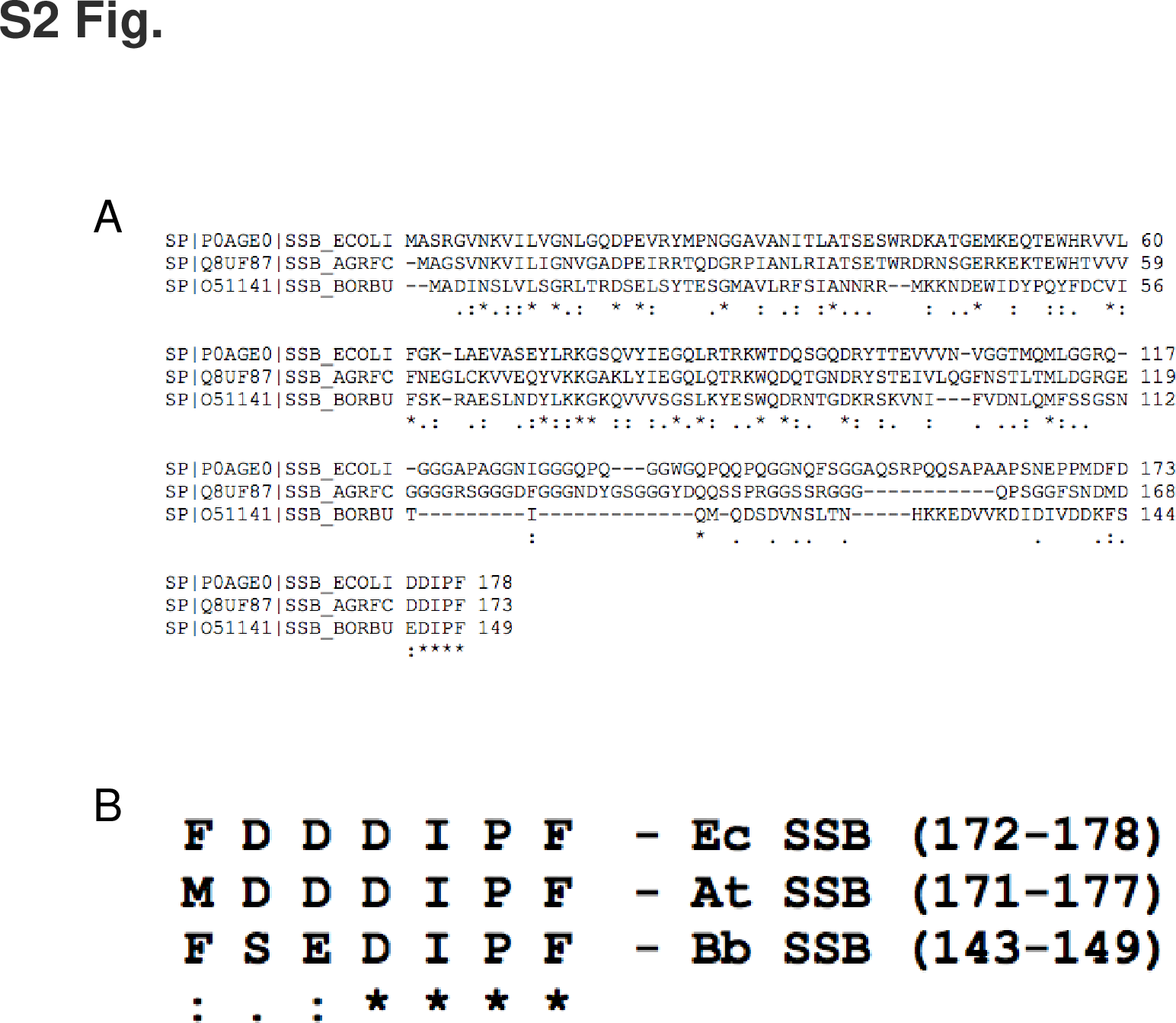

Supplement: S2 Fig — A) A multiple sequence alignment comparing SSB proteins from Escherichia coli, Agrobacterium tumefaciens, and Borrelia burgdorferi. Accession numbers: P0AGE0, SSB from Escherichia coli; O51141, SSB from Borrelia burgdorferi; Q8UF87, SSB from Agrobacterium tumefaciens. Identical amino acids are denoted by asterisks (*) and similar side chains by two dots (:). B) The conserved C-terminal tail identified in Escherichia coli (EcSSB) and Borrelia burgdorferi (BbSSB) SSBs is also present in Agrobacterium tumefaciens SSB (AtSSB). They share 100% similarity with one another in respect to their last seven amino acids. AtSSB shares stronger identity with EcSSB (85.7%) than BbSSB does at these positions. (TIF) [file pone.0246212.s002.tif]

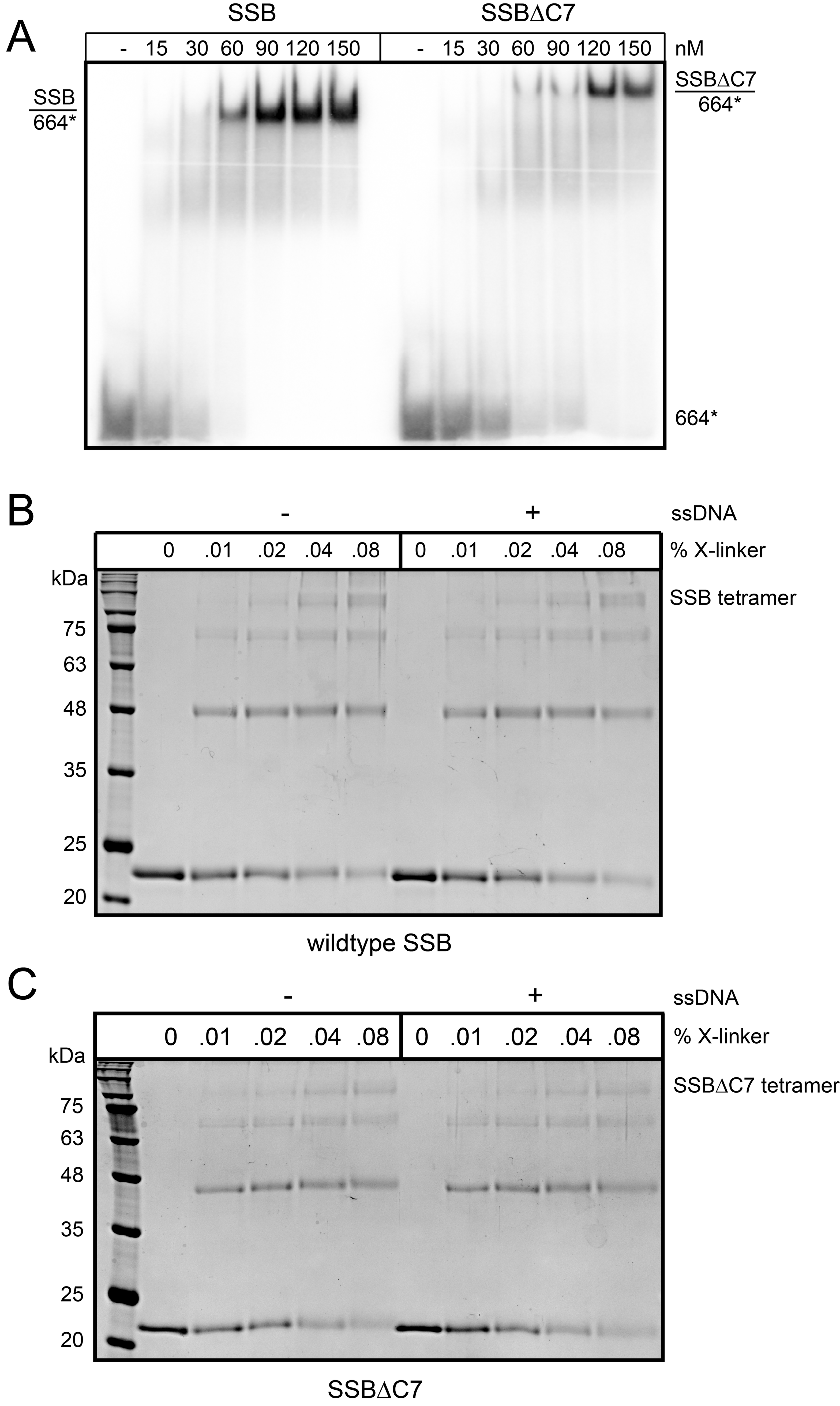

Supplement: S3 Fig — A) 8% PAGE/ 1X TAE gel of an electrophoretic mobility shift assay of wildtype AtSSB or AtSSBΔC7 with a 35-nt ssDNA reporter, OGCB664. Binding reactions contained 15 nM of 5’-32P endlabeled OGCB664 in 25 mM HEPES (pH 7.6), 0.1 mM EDTA and 50 mM potassium glutamate with variant concentrations of SSB as indicated in the key above the gel. Binding reactions were performed at 20°C for 10 min prior to reaction termination with loading dye containing no SDS to a 1X final concentration. B) Coomassie stained 5%/13% SDS-PAGE gel of AtSSB protein-protein crosslinking with or without unlabeled OGCB664 ssDNA. These reactions were used to assess the oligomeric status of AtSSB in solution and bound to ssDNA. 7.8 μM of AtSSB was incubated with or without 975 nM nucleotides of OGCB664 in 25 mM HEPES (pH 7.6), 0.1 mM EDTA and 160 mM potassium glutamate for 10 min at 20°C. Crosslinking was induced by addition of glutaraldehyde to final concentrations as indicated in the key above the gel followed by continued incubation at 20°C for 5 min. Excess crosslinker was quenched with Tris (pH 8.5) to a final concentration of 100 mM and additional incubation at 20°C for 5 min prior to gel loading. C) Coomassie stained 5%/13% SDS-PAGE gel of AtSSBΔC7 protein-protein crosslinking with or without unlabeled OGCB664 ssDNA. These reactions were used to assess the oligomeric status of AtSSBΔC7 in solution and bound to ssDNA. Reaction conditions are as reported in B). (TIF) [file pone.0246212.s003.tif]

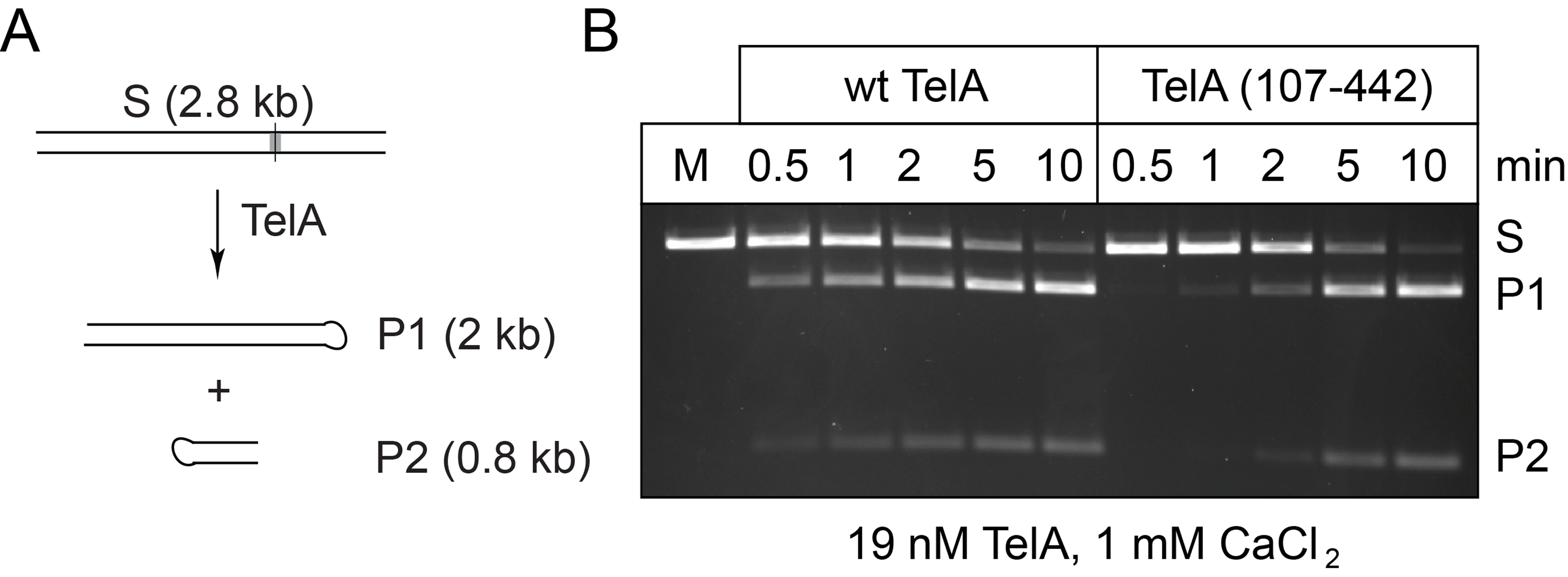

Supplement: S4 Fig — A) Schematic of the telomere resolution assay with a plasmid substrate. A pUC19 plasmid containing a 36 bp rTel sequence (grey, shaded area) was linearized by SspI. 1.75 μg/mL of the linearized plasmid (S) was combined with buffer containing 25 mM HEPES (pH 7.6), 1 mM DTT, 1 mM CaCl2, 100 μg/mL BSA, and 50 mM potassium glutamate. 19 nM of either wt TelA or TelA (107–442) was added to the reactions and incubated at 30°C. The conversion of (S) into two hairpin products (P1 and P2) was monitored by the removal of aliquots of the reaction mixture at indicated timepoints and combining them with SDS loading dye to 1X final concentration. B) Ethidium bromide stained 0.8% agarose/ 1X TAE gel panels showing telomere resolution timecourses with 19 nM of wt TelA and TelA (107–442). (TIF) [file pone.0246212.s004.tif]

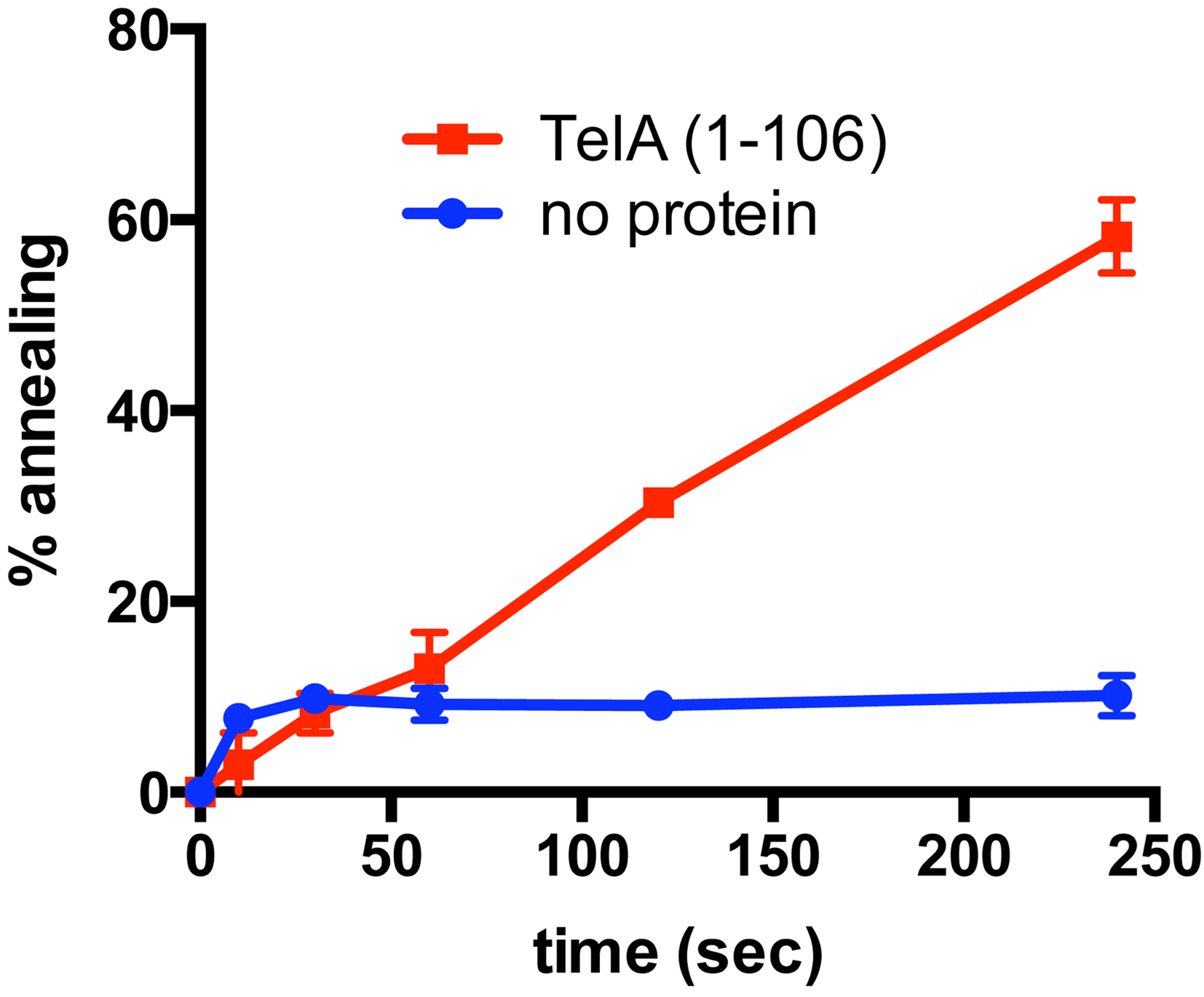

Supplement: S5 Fig — A plot of annealing timecourses comparing spontaneous annealing and annealing with 385 nM of TelA (1–106) with TAR substrates. The mean and standard deviation are shown and are derived from three separate experiments. (TIF) [file pone.0246212.s005.tif]

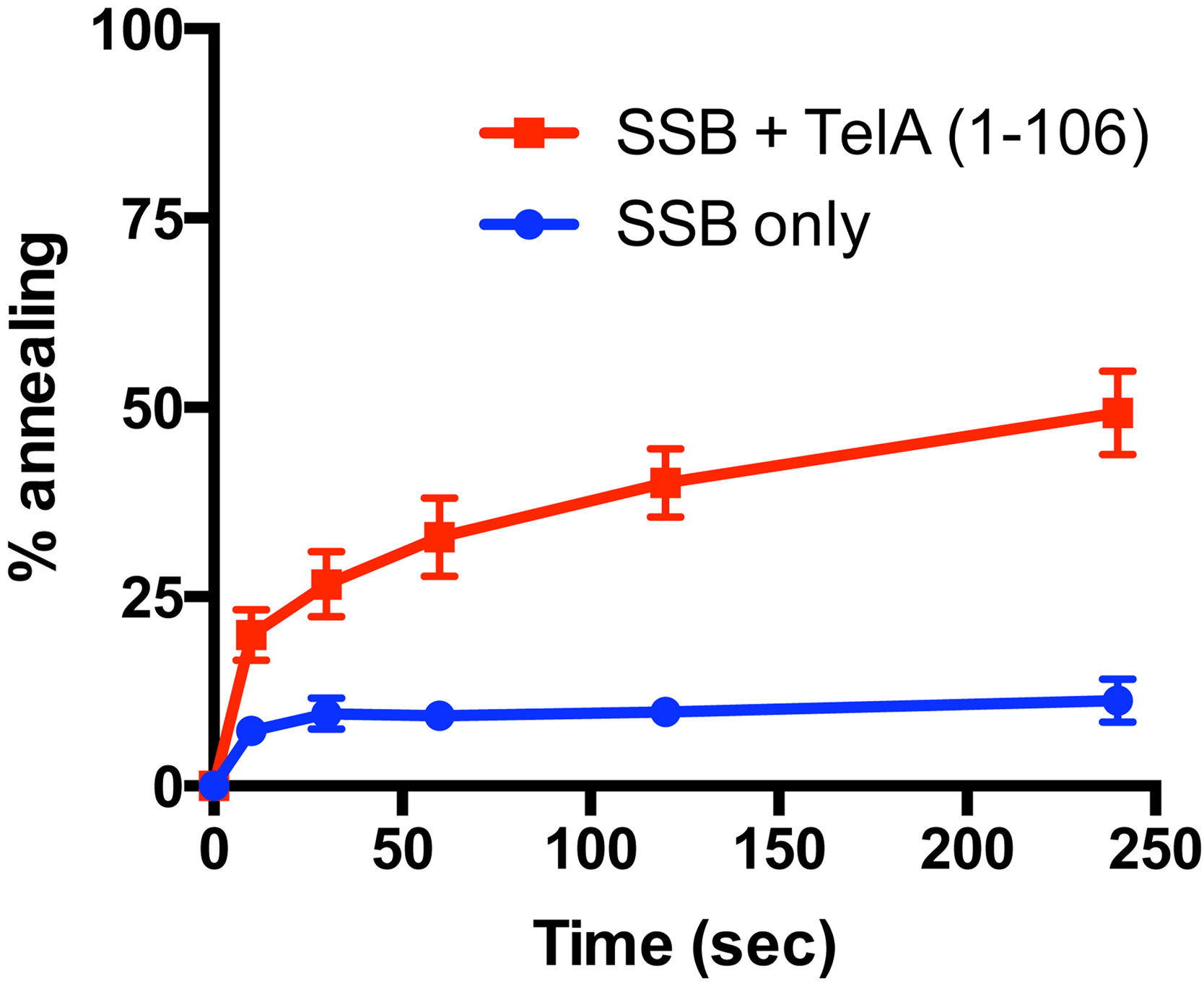

Supplement: S6 Fig — Plots of annealing timecourses with SSB-complexed DNA comparing reactions with and without 385 nM of TelA (1–106). SSB was present at 105 nM. (TIF) [file pone.0246212.s006.tif]

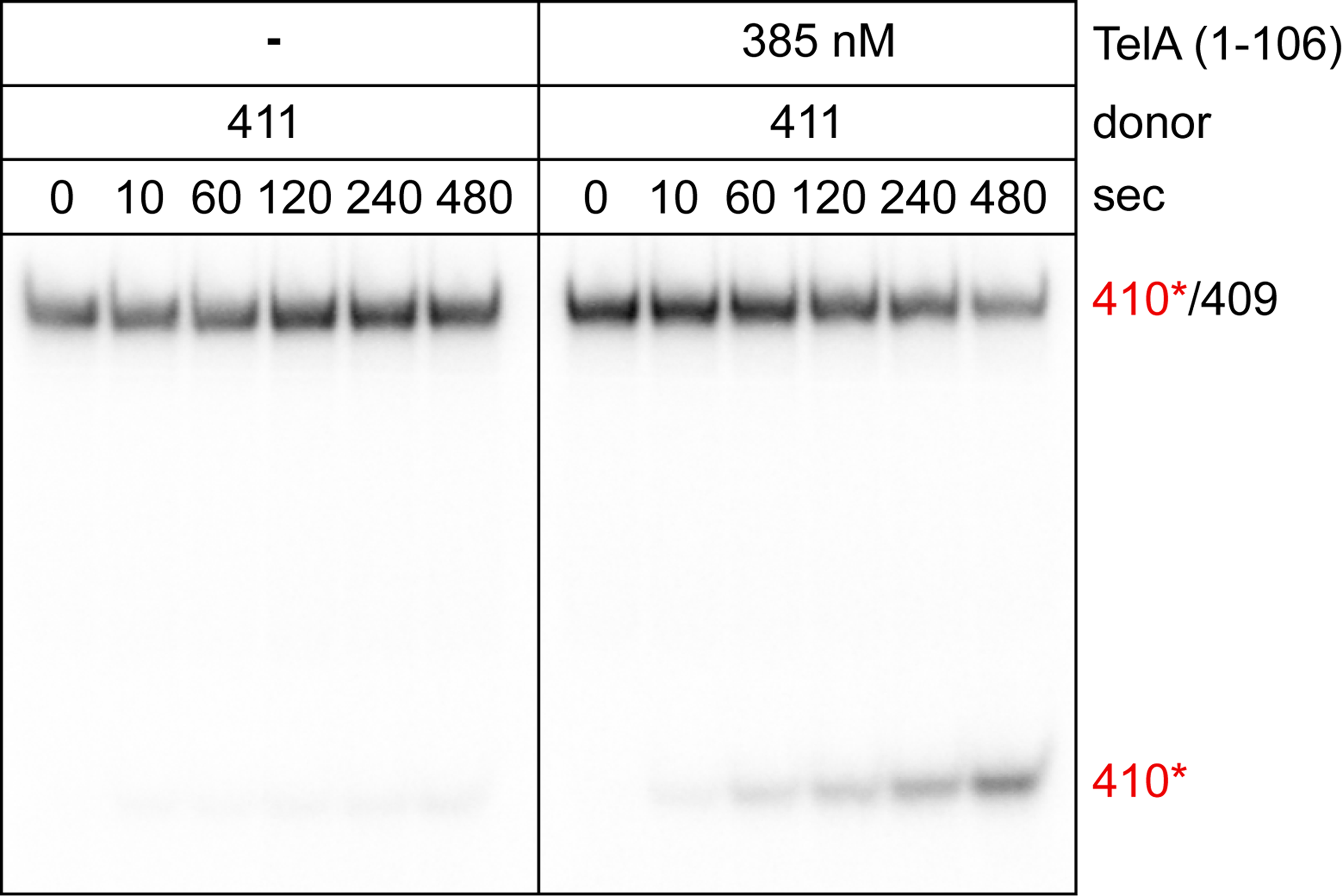

Supplement: S7 Fig — Representative 8% PAGE/ 1X TAE/ 0.1% SDS gel analysis of timecourse strand exchange reactions with and without TelA (1–106) present (385 nM). The migration position of the partial duplex is labelled as 410*/409 and the migration of the displaced strand if strand exchange occurs is labelled as 410*. (TIF) [file pone.0246212.s007.tif]

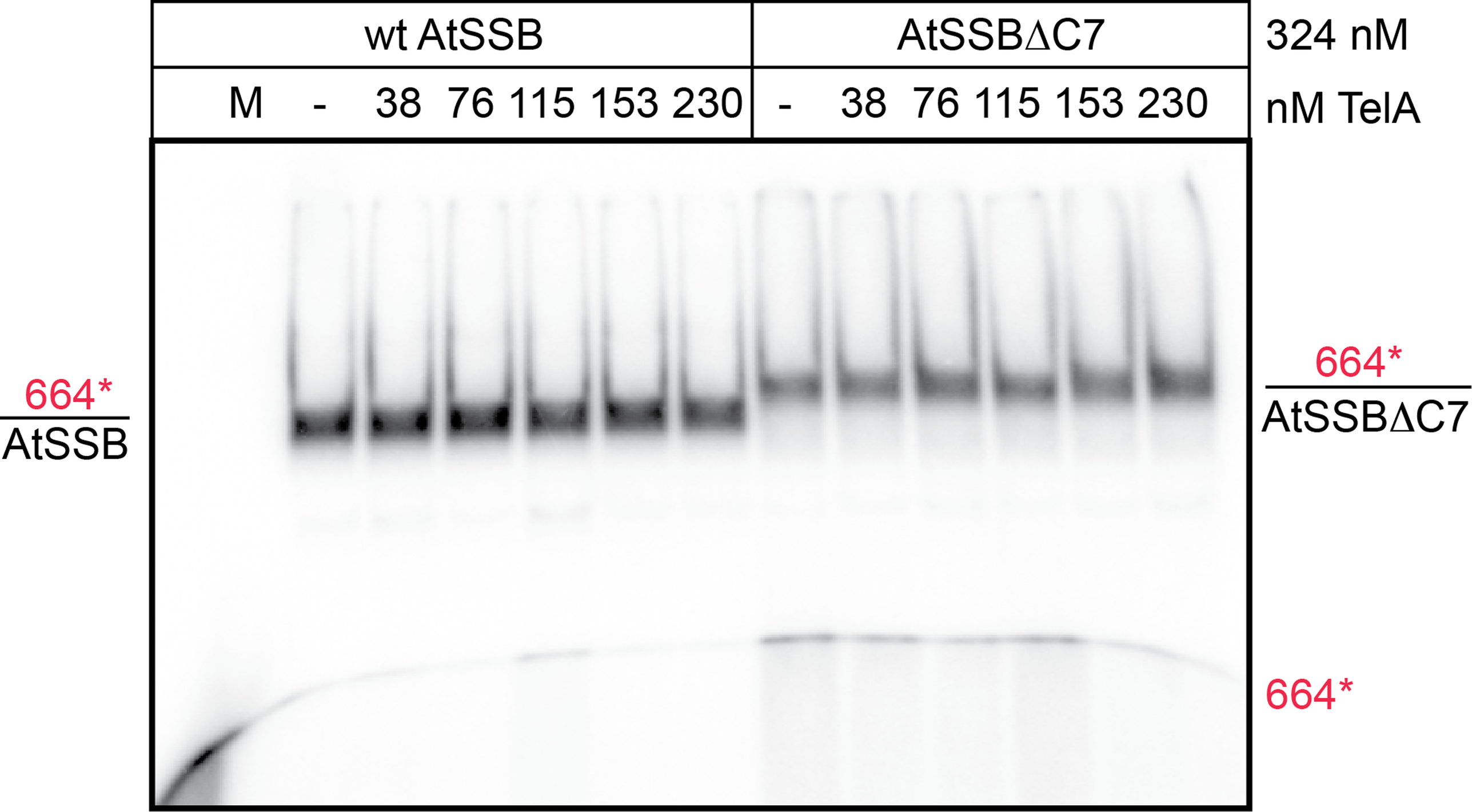

Supplement: S8 Fig — 7% PAGE/ 0.5X TBE gel of an electrophoretic mobility (co)shift assay of TelA with either AtSSB or AtSSBΔC7 and a 5’ radiolabeled 35-nt oligonucleotide (664*). AtSSB or AtSSBΔC7 was preincubated at 20°C for 10 min with 15 nM 664* in buffer containing 25 mM HEPES (pH 7.6), 1 mM DTT, 2 mM CaCl2, and 40 mM potassium glutamate. TelA was then added in a range of concentrations, as indicated in the legend, and the salt concentration adjusted to 100 mM potassium glutamate. The reactions were then further incubated for an additional 10 min prior to the addition of load dye containing no SDS. (TIF) [file pone.0246212.s008.tif]

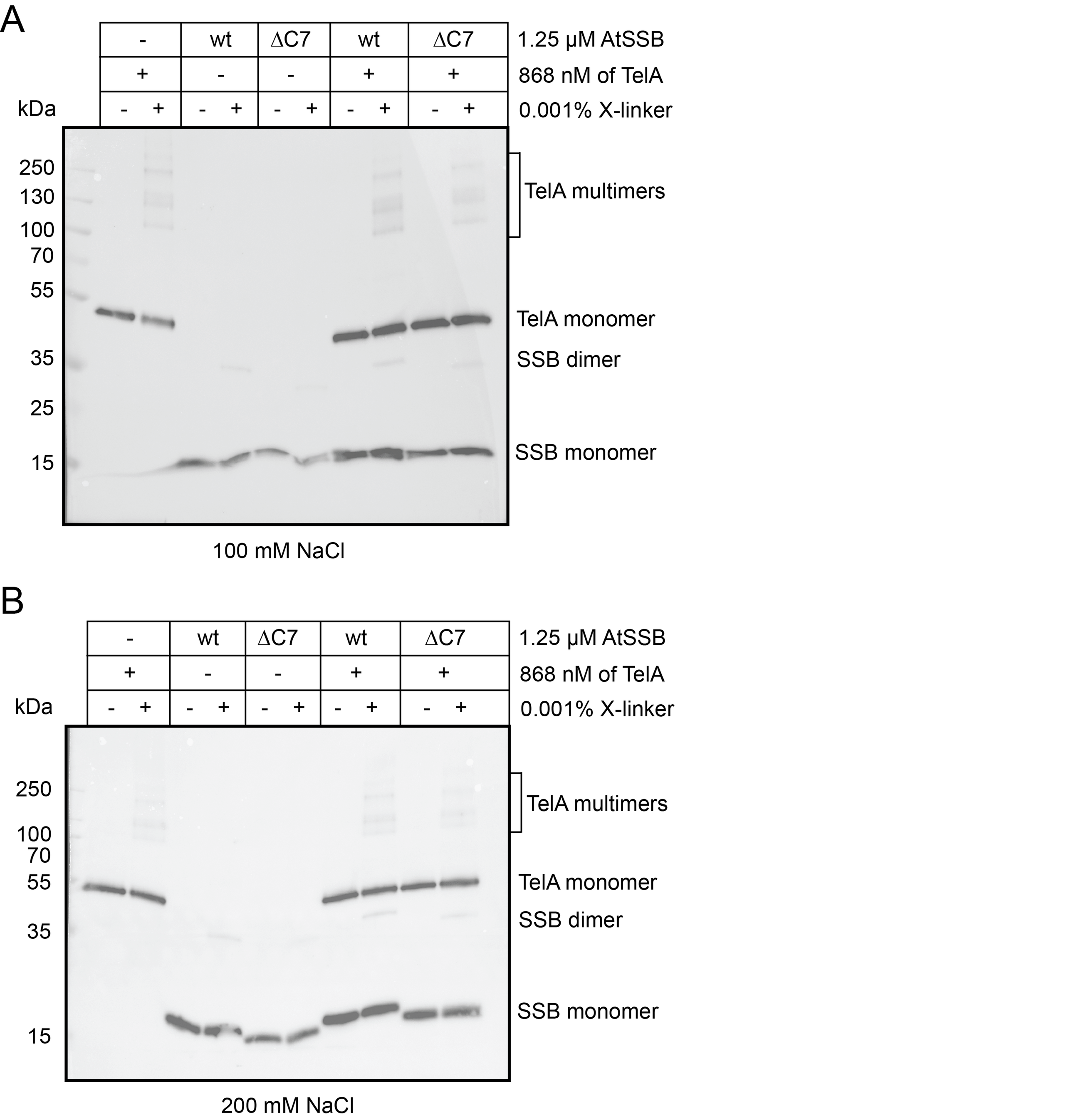

Supplement: S9 Fig — A) Protein-protein crosslinking performed in buffer containing 25 mM HEPES (pH 7.6), 0.1 mM EDTA, and 100 mM NaCl. TelA, AtSSB, and AtSSBΔC7 were present as indicated in the legend. Reactions were incubated at 30°C for 15 min. Crosslinking was induced by glutaraldehyde to a final concentration of 0.0001% followed by further incubation for 5 min. Excess crosslinker was quenched with Tris (pH 8.5) to a final concentration of 100 mM and additional incubation at 20°C for 5 min prior to gel loading. The results were visualized by application to a 3.5%/ 4–15% SDS-PAGE gradient gel followed by western blotting with a monoclonal antibody that recognizes the N-terminal 6X His tag on TelA and the SSBs. B) Protein-protein crosslinking performed in buffer containing 200 mM NaCl. Otherwise as described in A). (TIF) [file pone.0246212.s009.tif]
