# Supplementary material for: Single stranded DNA annealing is a conserved activity of telomere resolvases
Source: PLoS One. 2021 Feb 4;16(2):e0246212. doi: 10.1371/journal.pone.0246212 (PMC7861564; doi:10.1371/journal.pone.0246212)
Supplement: S1 Table — For all listed primers, the codon highlighted in red indicates the amino acid being altered from the parental strand. (DOCX) [file pone.0246212.s010.docx]

**S1 Table.**

| **Oligo name** | **Oligonucleotide sequence** | **Use** |
| --- | --- | --- |
| OGCB120 | 5’-GATCATTCTATACTAATTAAAAATTATAT ATATAATATTTATTTAGTATAAAGTgcaatggttagagga-3’ | 69 nt, used in ssDNA length requirements assay. Full complement of OGCB121 |
| OGCB121 | 5’-GATCtcctctaaccattgcACTTTATACTAAATAAATATTATAT ATATAATTTTTAATTAGTATAGAAT-3' | 69 nt, radiolabelled reporter oligo for ssDNA length requirements assay |
| OGCB302 | 5’-GATCATTCTATACTAATTAAAAATTAATA TATTAATATTTATTTAGTATAAAGTgcaatggttagagga-3’ | Used in ssDNA length requirements assay. Oligo 2 – possesses 6 nt block of non-complementary sequence with OGCB121 |
| OGCB392 | 5’-GATCATTCTATACTAATTAAAAAAATATA TATATTTATTTATTTAGTATAAAGTgcaatggttagagga-3’ | Used in ssDNA length requirements assay. Oligo 3 – possesses 12 nt block of non-complementary sequence with OGCB121 |
| OGCB393 | 5’-GATCATTCTATACTAATTAATTTAATATA TATATTATATTATTTAGTATAAAGTgcaatggttagagga-3’ | Used in ssDNA length requirements assay. Oligo 4 – possesses 18 nt block of non-complementary sequence with OGCB121 |
| OGCB403 | 5’-GATCATTCTATACTAATATTTTTAATATA TATATTATAAATTTTAGTATAAAGTgcaatggttagagga-3’ | Used in ssDNA length requirements assay. Oligo 5 – possesses 24 nt block of non-complementary sequence with OGCB121 |
| OGCB404 | 5’-GATCATTCTATACTTTAATTTTTAATATA TATATTATAAATAAAAGTATAAAGTgcaatggttagagga-3’ | Used in ssDNA length requirements assay. Oligo 6 – possesses 30 nt block of non-complementary sequence with OGCB121 |
| OGCB405 | 5’-GATCATTCTATTGATTAATTTTTAATATA TATATTATAAATAAATCAATAAAGTgcaatggttagagga-3’ | Used in ssDNA length requirements assay. Oligo 7 – possesses 36 nt block of non-complementary sequence with OGCB121 |
| OGCB406 | 5’-GATCATTCATATGATTAATTTTTAATATA TATATTATAAATAAATCATATAAGTgcaatggttagagga-3’ | Used in ssDNA length requirements assay. Oligo 8 – possesses 42 nt block of non-complementary sequence with OGCB121 |
| OGCB409 | 5’-TCTGCGCCTCGTTCCGGCTA**AGTAACATGGAGCAGGTCGCGGATTTCGAC**  **ACAATTTATCAGG**CGATGATACAAATCTCCGTT**GTACTTTGTTTCGCGCTTGGTATAATCGCTGGGGGTCAAAGAT-3’** | 126 nt, anneal with OGCB410 for SE assays |
| OGCB410 | 5’-**CCTGATAAATTGTGTCGAAATCCGCGACCTGCTCCATGTTACT**-3’ | 43 nt, anneal with OGCB409 for SE assays |
| OGCB411 | 5’-**CCTGATAAATTGTGTCGAAATCCGCGACCTGCTCCATGTTACT**  TAGCCGGAACGAGGCGCAGA-3’ | 63 nt, donor strand for SE assays. 20 nt 3’ homology to OGCB409 |
| OGCB426 | 5’-(N)_63_-3’ | Random 63 nt. Used as a control donor in SE assays |
| OGCB455 | 5’-GATCATATCCTTTCTTTAAACTTCTATCATTGATTCTTACTAG TCTTTACCTTACTATACTTCTATCAGTTTATCGATTCTTCTTTA-3’ | 87 nt ssDNA; used in annealing assays. Use with OGCB456. |
| OGCB456 | 5’-GATCTAAAGAAGAATCGATAAACTGATAGAAGTATAGTAAG GTAAAGACTAGTAAGAATCAATGATAGAAGTTTAAAGAAAGGATAT-3’ | Complement of 455 to make a duplex DNA in annealing assays. |
| OGCB478 | 5’-gatcTAAGATATGATTAATTTTTAATATA TATATTATAAATTTTAGTATAAAGTgcaatggttagagga-3’ | Marker for mobilty of frayed-end product. Annealed with OGCB121 |
| OGCB664 | 5’-GGAAGCGATAAAACTCTGCAGGTTGGATACGCCAA-3’ | 35-nt ssDNA oligo used in SSB EMSA and crosslinking experiments, Fig. S3 |
| OGCB666 | 5’-GTTACTTAGCCGGAACGAGGCGCAGA-3’ | 26 nt strand to construct 3’-partial duplex substrate used for nuclease testing. Use with OGCB692 |
| OGCB692 | 5’-TCTGCGCCTCGTTCCGGCTAAGTAACATGGAGCAGGTC GCGGATTTCGACACAATTTATCAGGCGATGATACAAAT-3’ | 76 nt strand to make 3’-partial duplex used for nuclease testing. Use with OGCB666. |
| OGCB794 | 5’-**ATACCGGATCC**TTACCCCTTACGACTTACGGGCGC-3’ | Primer for mutant plasmid construct of TelA (107-442). Use with OGCB795 |
| OGCB795 | 5’-**CACCATGGCATATG**GGAGTGGCGACCTCTATCGTTGAAAAG-3’ | Primer for mutant plasmid construct of TelA (107-442). Use with OGCB794. |
| OGCB778 | 5’-TTAGAAACAAGTCTTTCTTTCATGACTTATACGCTGCCT-3’ | Primer for mutant plasmid construct of TelA (Y405F). Use with OGCB779. |
| OGCB779 | 5’-AGGCAGCGTATAAGTCATGAAAGAAAGACTTGTTTCTAA-3’ | Primer for mutant plasmid construct of TelA (Y405F). Use with OGCB778. |
| OGCB796 | 5’-**CACCATGGCAT**ATGGCTGGCTCAGTAAATAAGGTAATTTTAAT  AGGTAAC-3’ | Primer for mutant plasmid construct of agrobacterium SSBΔC7. Use with OGCB797. |
| OGCB797 | 5’-**ATACCGGATCC**TTAATCGTTGCTAAAGCCCCCACTCGG-3’ | Primer for mutant plasmid construct of agrobacterium SSBΔC7. Use with OGCB796. |
| OGCB876 | 5’-**CACCATGGCATATG**CTGGCGGCCAAGCGTAAGACA-3’ | Primer for mutant plasmid construct, TelA (1-106). Use with OGCB877 |
| OGCB877 | 5’-**ATACCGGATCC**TTAGGTTTTGGGATAATCGCCACGTGCAATATAT  GT-3’ | Primer for mutant plasmid construct, TelA (1-106). Use with OGCB876 |
| OGCB898 | 5’-GGTCTCTCTTGTTAGACCAGGTCGAGCCCGGGAGCTCTCTGGC  TAGCAAGGAACCC-3’ | HIV TAR sense TAR(+); use with 899 for annealing assays |
| OGCB899 | 5’-GGGTTCCTTGCTAGCCAGAGAGCTCCCGGGCTCGACCTGGTC  TAACAAGAGAGACC-3’ | HIV TAR sense TAR(-); use with 898 for annealing assays |
