# Supplementary material for: Single stranded DNA annealing is a conserved activity of telomere resolvases
Source: PLoS One. 2021 Feb 4;16(2):e0246212. doi: 10.1371/journal.pone.0246212 (PMC7861564; doi:10.1371/journal.pone.0246212)
Supplement: S1 Sequence — The gene sequence and corresponding amino acid sequence for the synthetic TelA gene are shown and numbered, respectively. The NdeI and BamHI restriction sites are bolded and the stop codon introduced into the synthetic gene is highlighted in red. The synthetic gene was blunt-end cloned into pUCIDT by IDT and verified by DNA sequencing. This figure was generated from https://www.bioinformatics.nl/cgi-bin/emboss/prettyseq. (DOCX) [file pone.0246212.s011.docx]

**S1 sequence.**

1 **catatg**ctggcggccaagcgtaagacaaaaacgccagtgttagtggaacgtatcgaccaattcgtcggtcaaattaaagaa 81

1 M L A A K R K T K T P V L V E R I D Q F V G Q I K E 27

82 gccatgaaatcggacgatgctagtcgcaatcgcaaaatccgtgatctgtgggatgcggaggtgcgctatcatttcgacaac 162

28 A M K S D D A S R N R K I R D L W D A E V R Y H F D N 54

163 ggtcgtactgaaaagaccttggagttatacattatgaaatatcgtaatgcattaaaagccgaatttggacccaagtcaaca 243

55 G R T E K T L E L Y I M K Y R N A L K A E F G P K S T 81

244 ccgctggctatctgtaatatgaagaagttgcgcgagcgcctgaacacatatattgcacgtggcgattatcccaaaaccgga 324

82 P L A I C N M K K L R E R L N T Y I A R G D Y P K T G 108

325 gtggcgacctctatcgttgaaaagatcgagcgtgccgagttcaataccgcgggccgtaaacctacagttttattgcgtatc 405

109 V A T S I V E K I E R A E F N T A G R K P T V L L R I 135

406 gcagacttcattgctgcaatgaacggtatggatgctaaacaagacatgcaggccctttgggacgccgaaattgctattatg 486

136 A D F I A A M N G M D A K Q D M Q A L W D A E I A I M 162

487 aacggccgtgctcagacaactatcatctcctacattacaaaataccgcaatgcgattcgcgaagcttttggggacgaccac 567

163 N G R A Q T T I I S Y I T K Y R N A I R E A F G D D H 189

568 ccaatgttgaaaattgccactggcgacgctgctatgtacgacgaagctcgtcgtgttaagatggaaaaaatcgcgaataaa 648

190 P M L K I A T G D A A M Y D E A R R V K M E K I A N K 216

649 cacggtgcacttatcacgtttgaaaattatcgccaggtcctgaaaatctgcgaggattgcttgaaaagctccgacccactt 729

217 H G A L I T F E N Y R Q V L K I C E D C L K S S D P L 243

730 atgatcgggattggccttattgggatgacggggcgtcgcccctacgaagtctttactcaagctgagtttagtccagctccg 810

244 M I G I G L I G M T G R R P Y E V F T Q A E F S P A P 270

811 tacggaaaaggggtatcgaagtggtcgatcctttttaacggacaggccaagactaagcaaggtgagggaacgaagtttggg 891

271 Y G K G V S K W S I L F N G Q A K T K Q G E G T K F G 297

892 atcacgtacgaaatccctgtcttgacccgctcagaaactgtccttgccgcctacaagcgcctgcgtgaaagtggccaaggc 972

298 I T Y E I P V L T R S E T V L A A Y K R L R E S G Q G 324

973 aagttgtggcatggcatgtcgatcgacgacttctcgtccgagacacgtctgctgttacgcgatacggtctttaacttgttt 1053

325 K L W H G M S I D D F S S E T R L L L R D T V F N L F 351

1054 gaggatgtttggccgaaggaagagcttccgaaaccgtacggtcttcgccacttgtacgctgaagtggcataccataatttt 1134

352 E D V W P K E E L P K P Y G L R H L Y A E V A Y H N F 378

1135 gcacctccgcacgtcactaaaaacagttatttcgcagccatcctgggccacaacaataatgacttagaaacaagtctttct 1215

379 A P P H V T K N S Y F A A I L G H N N N D L E T S L S 405

1216 tacatgacttatacgctgcctgaggaccgtgataacgcactggcgcgcctgaagcgcaccaacgaacgcacattgcaacag 1296

406 Y M T Y T L P E D R D N A L A R L K R T N E R T L Q Q 432

1297 atggccaccattgcgcccgtaagtcgtaagggg**taaggatcc** 1338

433 M A T I A P V S R K G
