# Supplementary material for: Single stranded DNA annealing is a conserved activity of telomere resolvases
Source: PLoS One. 2021 Feb 4;16(2):e0246212. doi: 10.1371/journal.pone.0246212 (PMC7861564; doi:10.1371/journal.pone.0246212)
Supplement: S2 Sequence — The gene sequence and corresponding amino acid sequence for the synthetic AtSSB gene are shown and numbered, respectively. The NdeI and BamHI restriction sites are bolded and the stop codon introduced into the synthetic gene is highlighted in red. The synthetic gene was blunt-end cloned into pUCIDT by IDT and verified by DNA sequencing. This figure was generated from https://www.bioinformatics.nl/cgi-bin/emboss/prettyseq. (DOCX) [file pone.0246212.s012.docx]

**S2 sequence.**

1 **catatg**gctggctcagtaaataaggtaattttaataggtaacgtaggagctgatccggagatacgccggacgcaagacggg 81

1 H M A G S V N K V I L I G N V G A D P E I R R T Q D G 27

82 cgcccaatagccaacttgcggatcgcaacaagcgaaacttggagagaccgtaatagcggagaacgtaaggaaaaaaccgaa 162

28 R P I A N L R I A T S E T W R D R N S G E R K E K T E 54

163 tggcacaccgttgtagtgtttaatgaaggtctttgcaaggtggttgagcagtacgttaagaaaggggcgaaattgtatatt 243

55 W H T V V V F N E G L C K V V E Q Y V K K G A K L Y I 81

244 gagggtcagctgcagactagaaagtggcaagatcagactggcaacgaccggtactcaaccgaaatagttttacaaggtttc 324

82 E G Q L Q T R K W Q D Q T G N D R Y S T E I V L Q G F 108

325 aacagcacgttgacaatgctggacgggcgtggggaggggggtgggggtcgtagcggcggcggtgactttggtggaggcaat 405

109 N S T L T M L D G R G E G G G G R S G G G D F G G G N 135

406 gattacgggtccggcgggggttatgaccagcaatcgtcgccaagaggggggagtagccggggtggcggacagccgagtggg 486

136 D Y G S G G G Y D Q Q S S P R G G S S R G G G Q P S G 162

487 ggctttagcaacgatatggatgatgacattcctttc**taaggatcc** 531

163 G F S N D M D D D I P F ***** 177
