# Supplementary material for: Single stranded DNA annealing is a conserved activity of telomere resolvases
Source: PLoS One. 2021 Feb 4;16(2):e0246212. doi: 10.1371/journal.pone.0246212 (PMC7861564; doi:10.1371/journal.pone.0246212)
Supplement: S1 Raw images — (PDF) [file pone.0246212.s013.pdf]

Fig 1 raw gels:

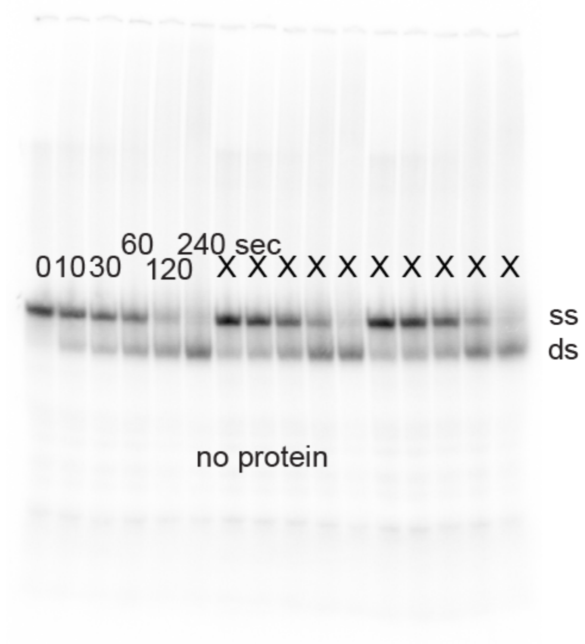

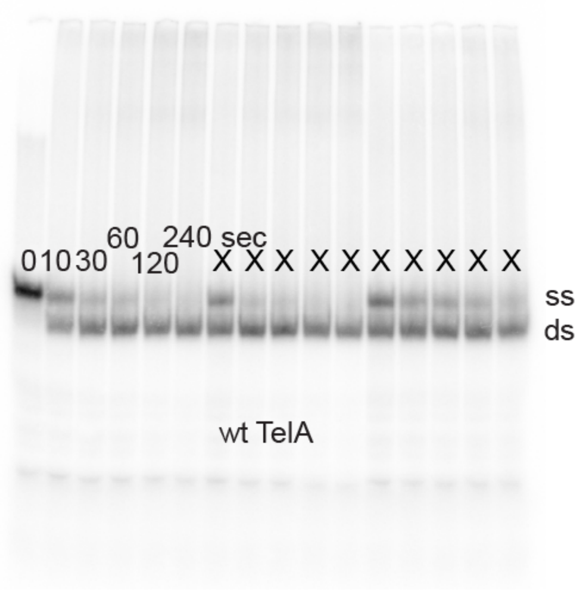

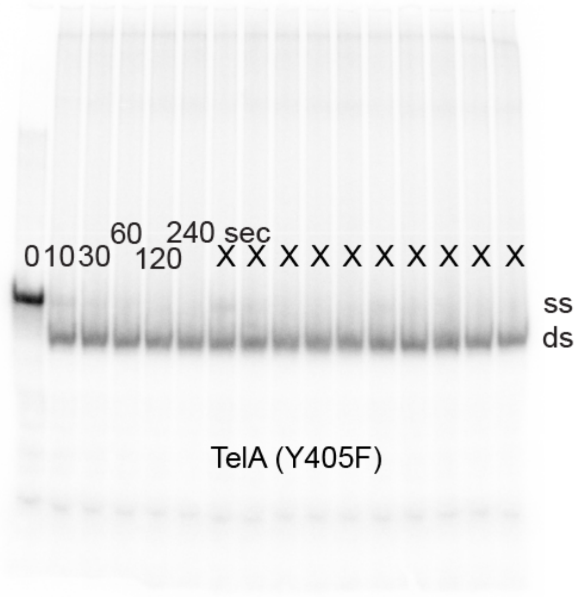

Gels with timecourse reactions, performed in triplicate, used in Fig 1. A single replicate was shown of each condition in Fig 1. X's indicate lanes from replicates not shown in the gel panels.

Fig 2 raw images:

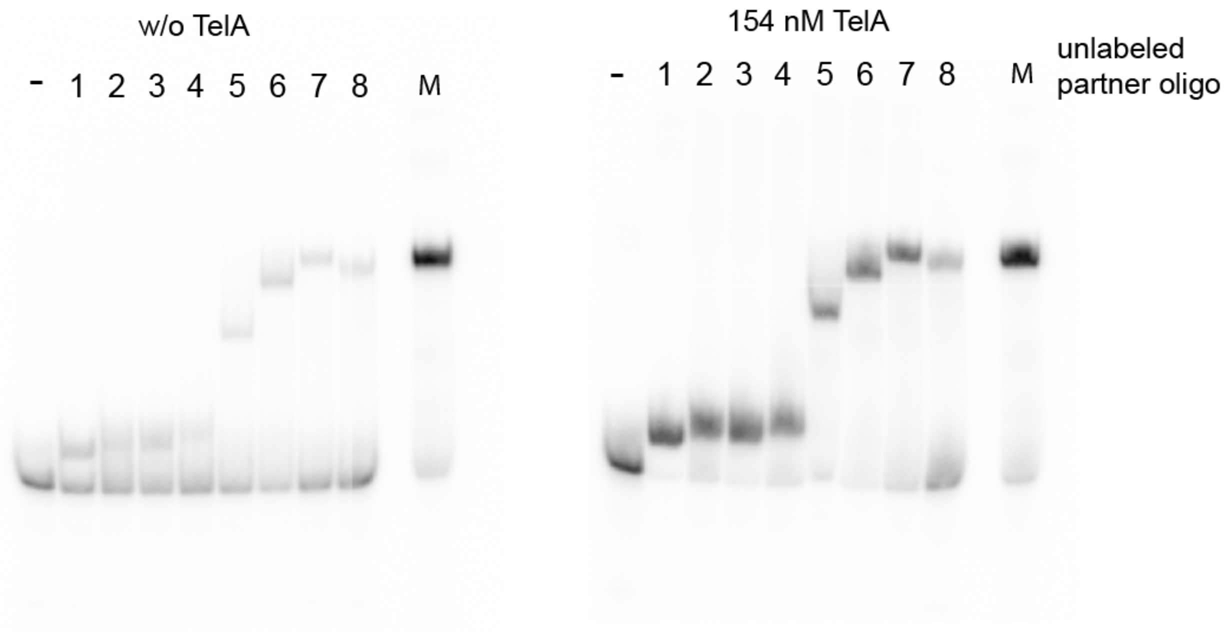

Gels for protein free and TelA annealing assays shown in Fig 2. M indicates the frayed ends marker used to determine the gel migration breakpoint bewteen bubble and frayed end products.

Fig 3 raw images:

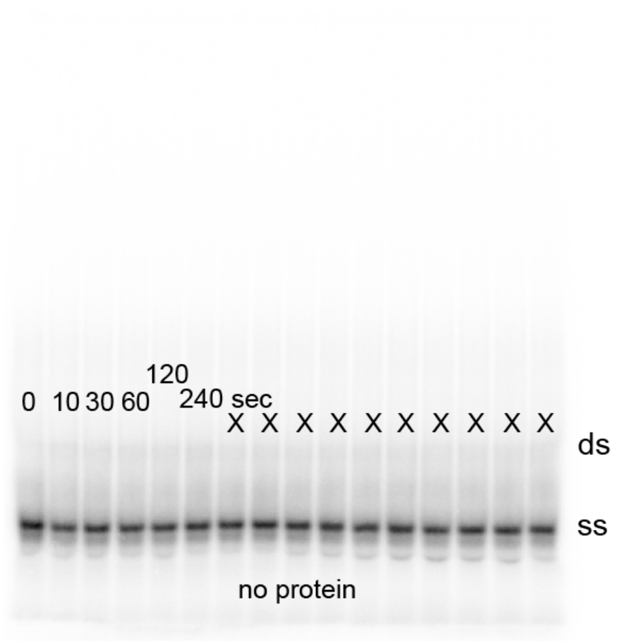

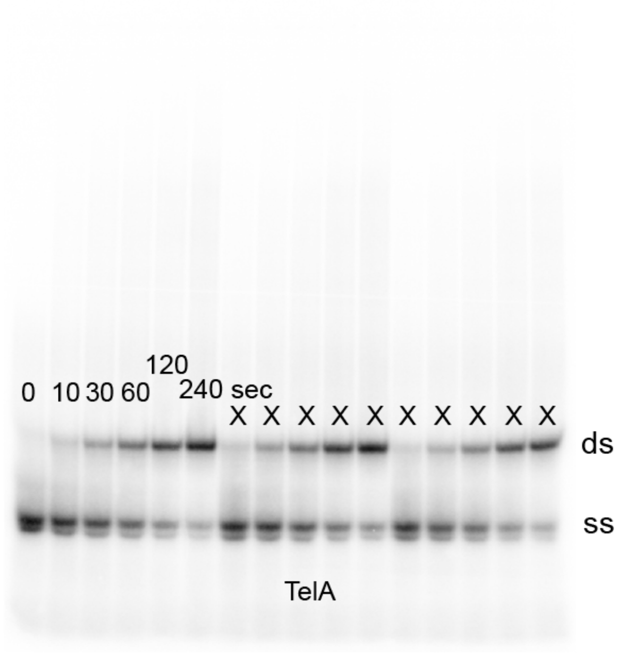

Gels for protein free and TelA annealing assays shown in Fig 3. X’s indicate lanes of replicates not shown in Fig 3.

Fig 4 raw images:

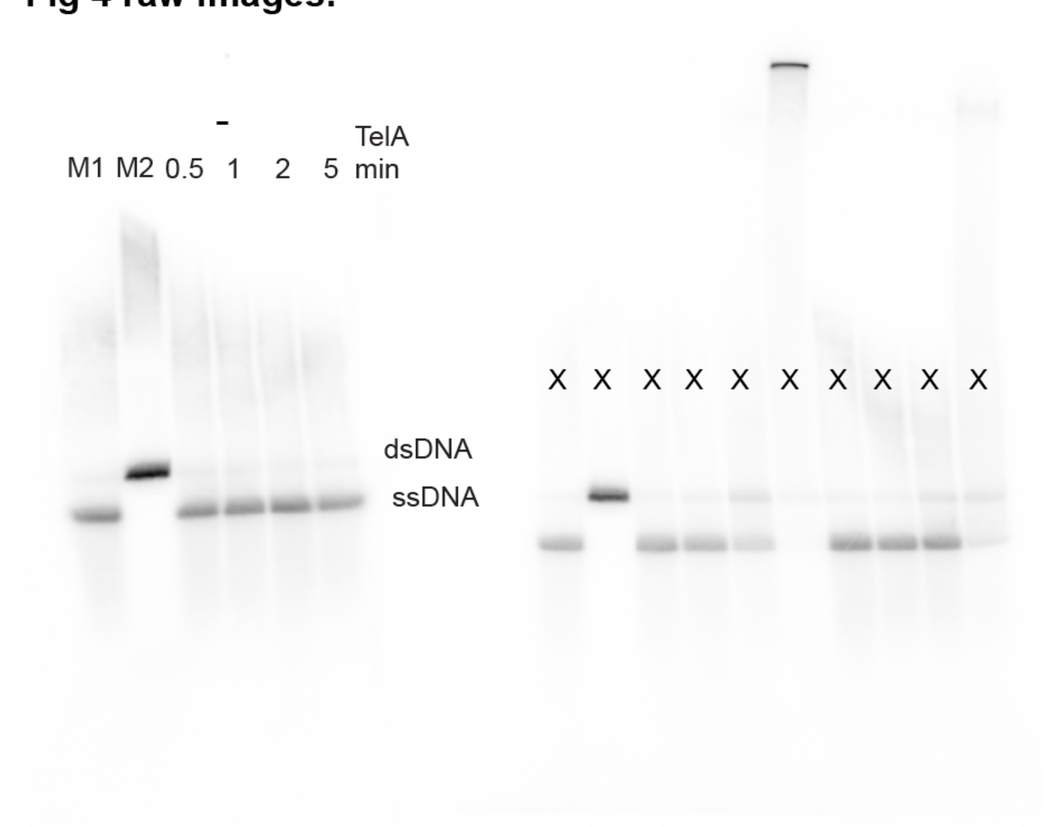

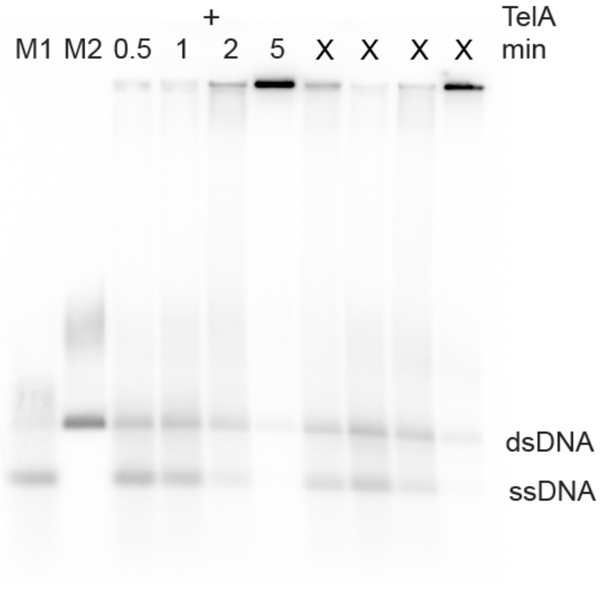

Gels for plasmid annealing assays shown in Fig 4. X's indicate lanes of conditions not shown in Fig 4.

Fig 5 raw images:

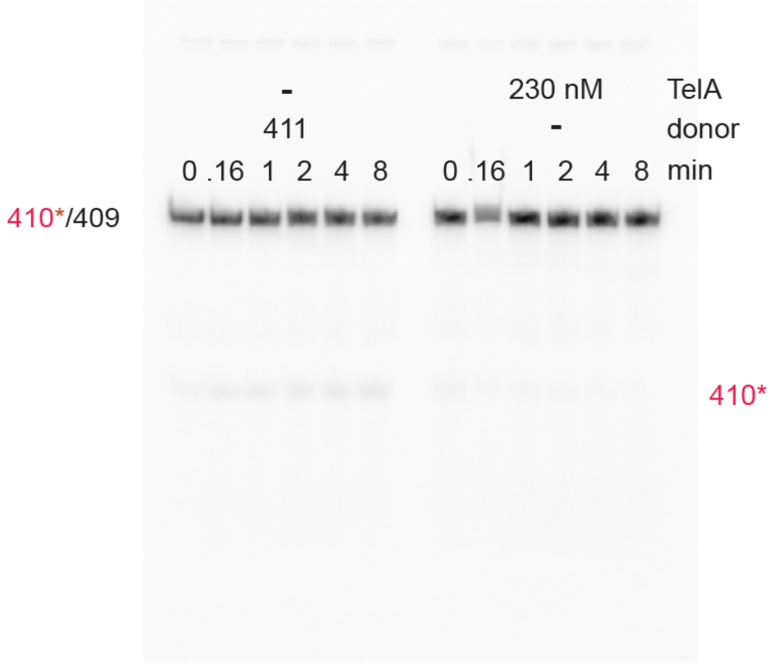

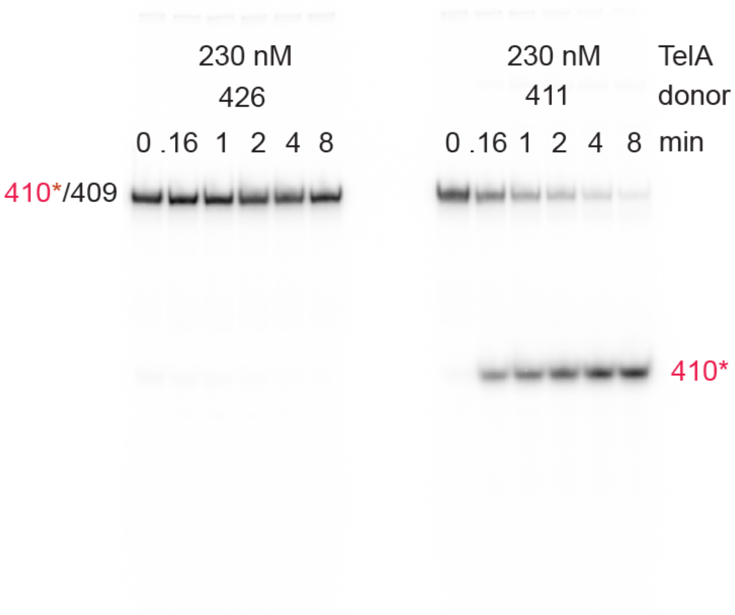

Gels for strand exchange assays shown in Fig 5.

Fig 7 raw images:

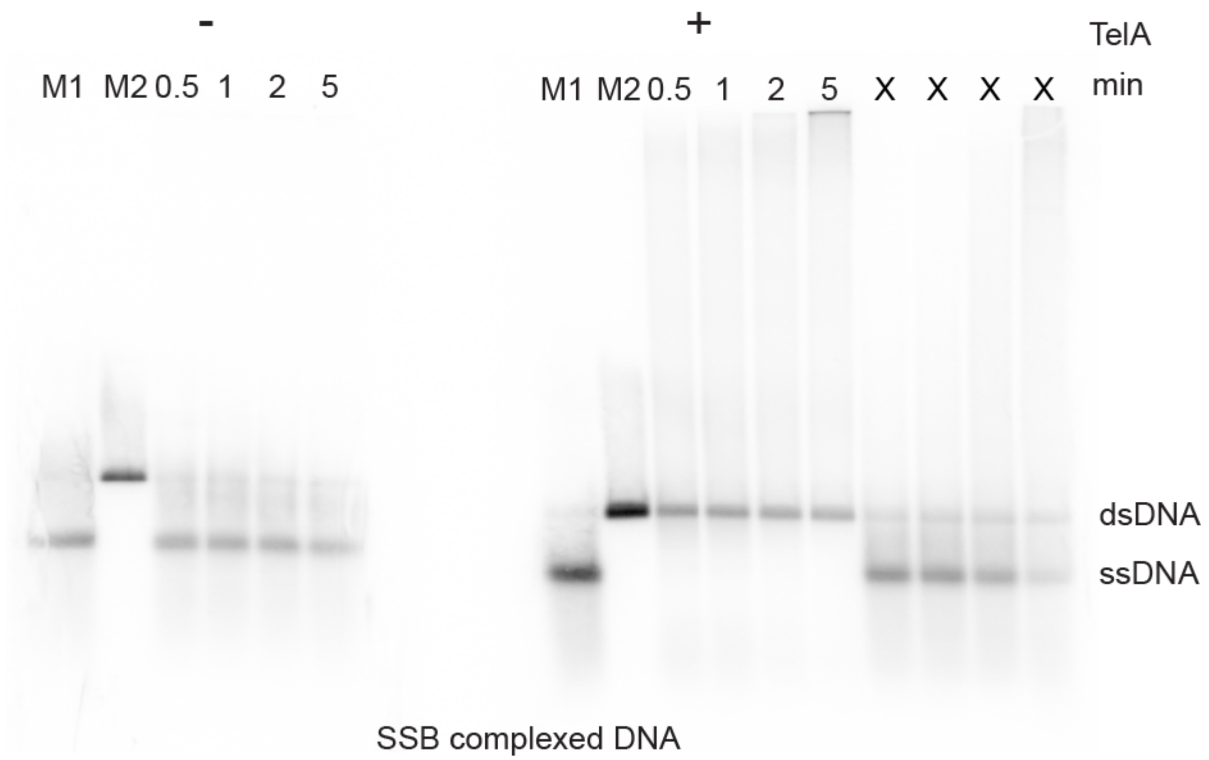

Gels for plasmid annealing assays shown in Fig 7. X’s indicate lanes of conditions not shown in Fig 7.

Fig 8 raw images:

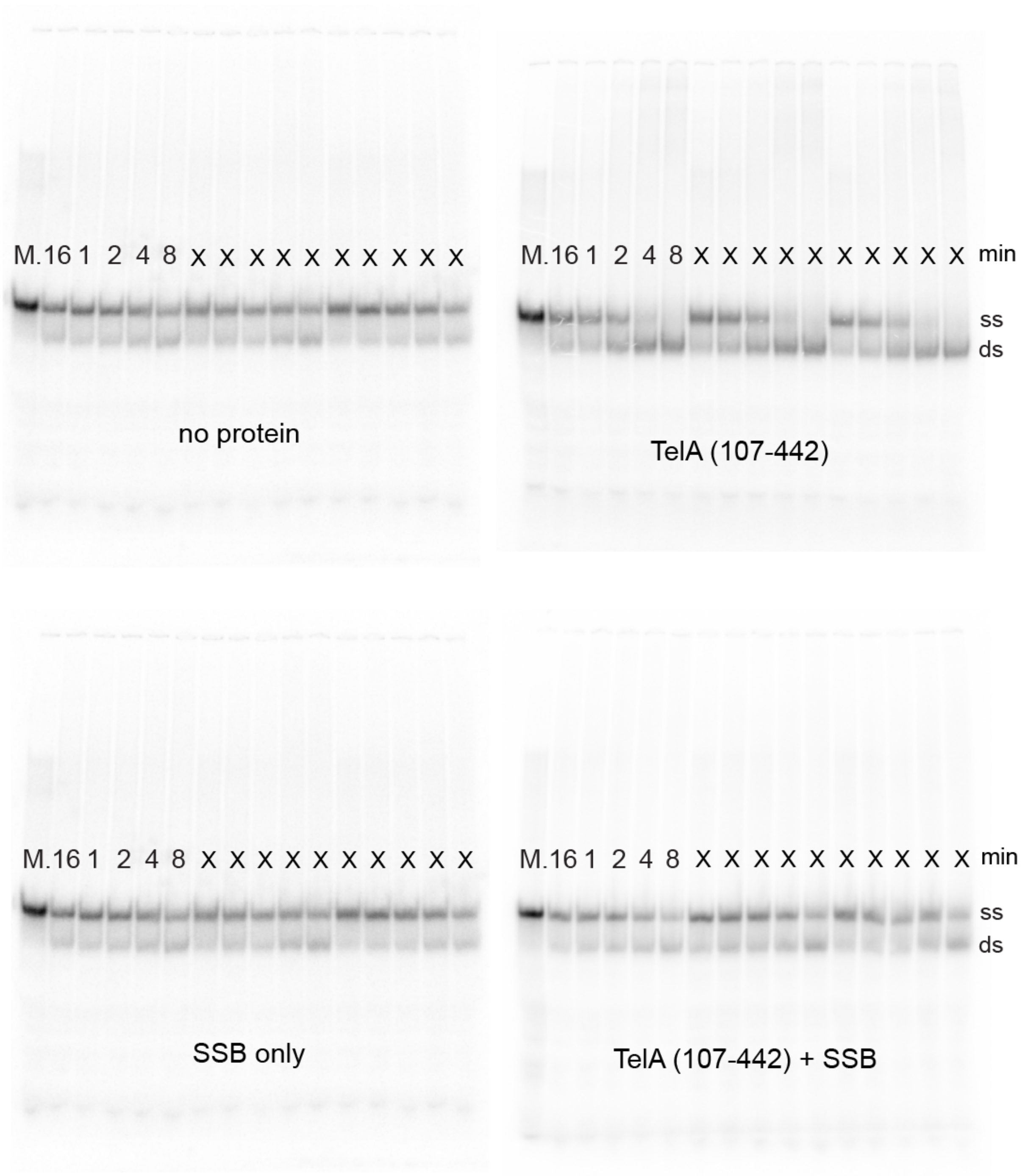

Gels with timecourse reactions, performed in triplicate, used in Fig 8A. A single replicate was shown of each condition in Fig 8A. X's indicate lanes of replicates not shown in Fig 8.

Fig 9 raw images:

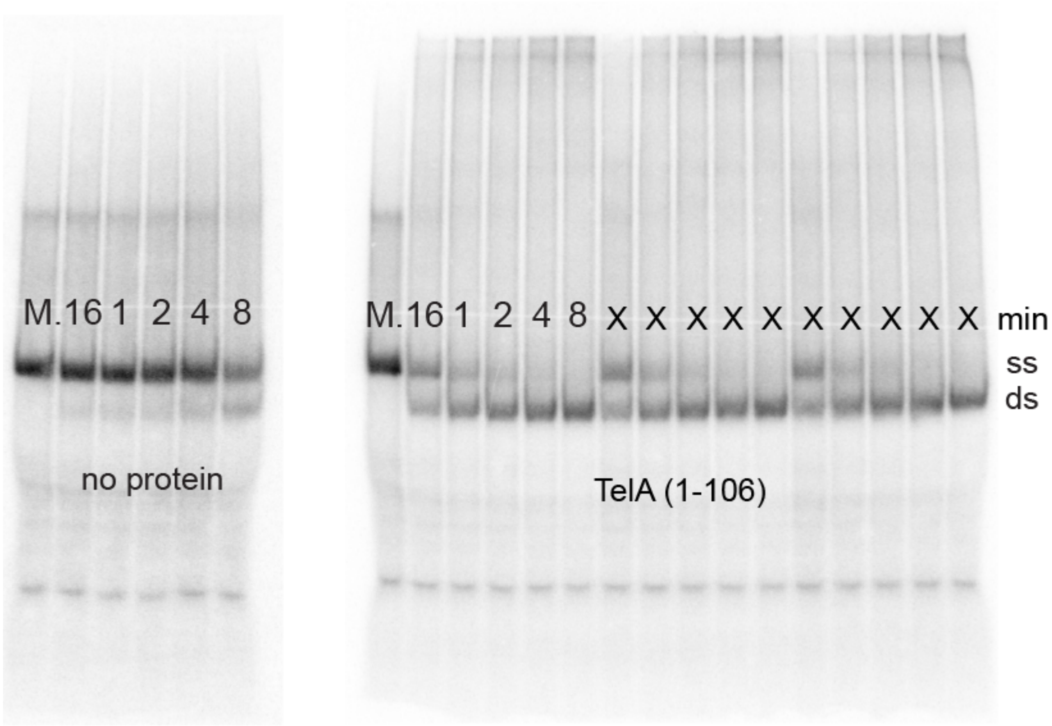

Gels for protein free and TelA annealing assays shown in Fig 9. X's indicate lanes of replicates not shown in Fig 9.

S1 Fig raw images:

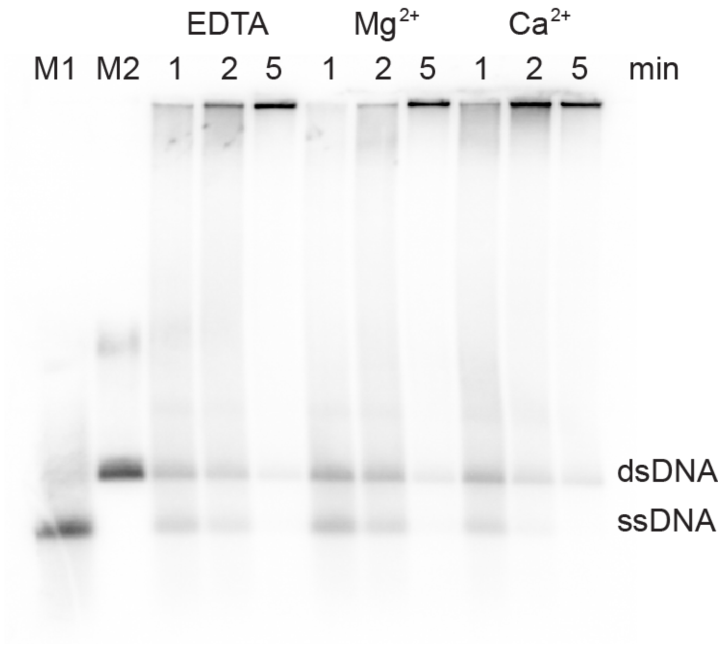

Gel for plasmid annealing assays shown in S1 Fig.

S3 Fig raw images:

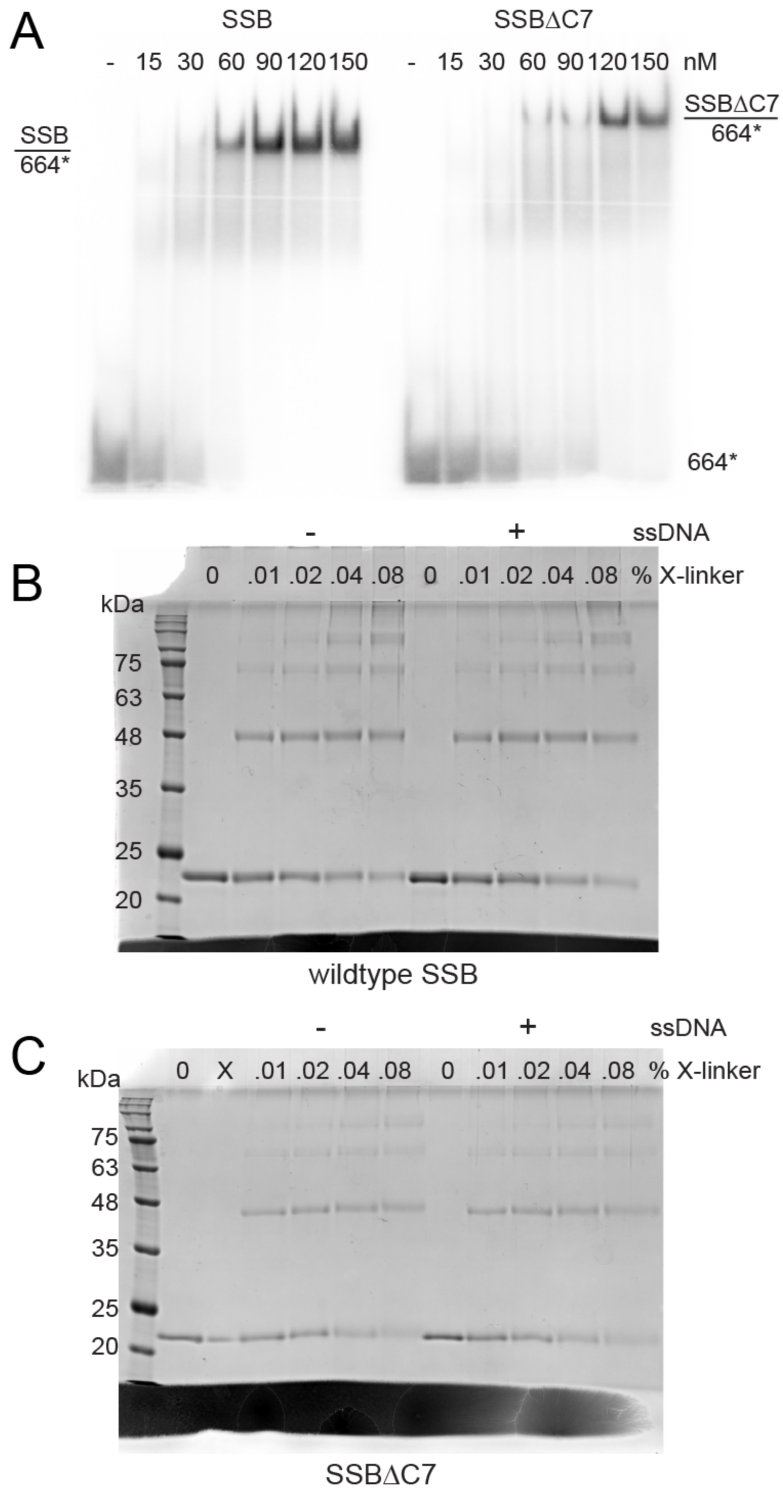

EMSA and Coomassie stained gels shown in S3 Fig.

S6 Fig raw images:

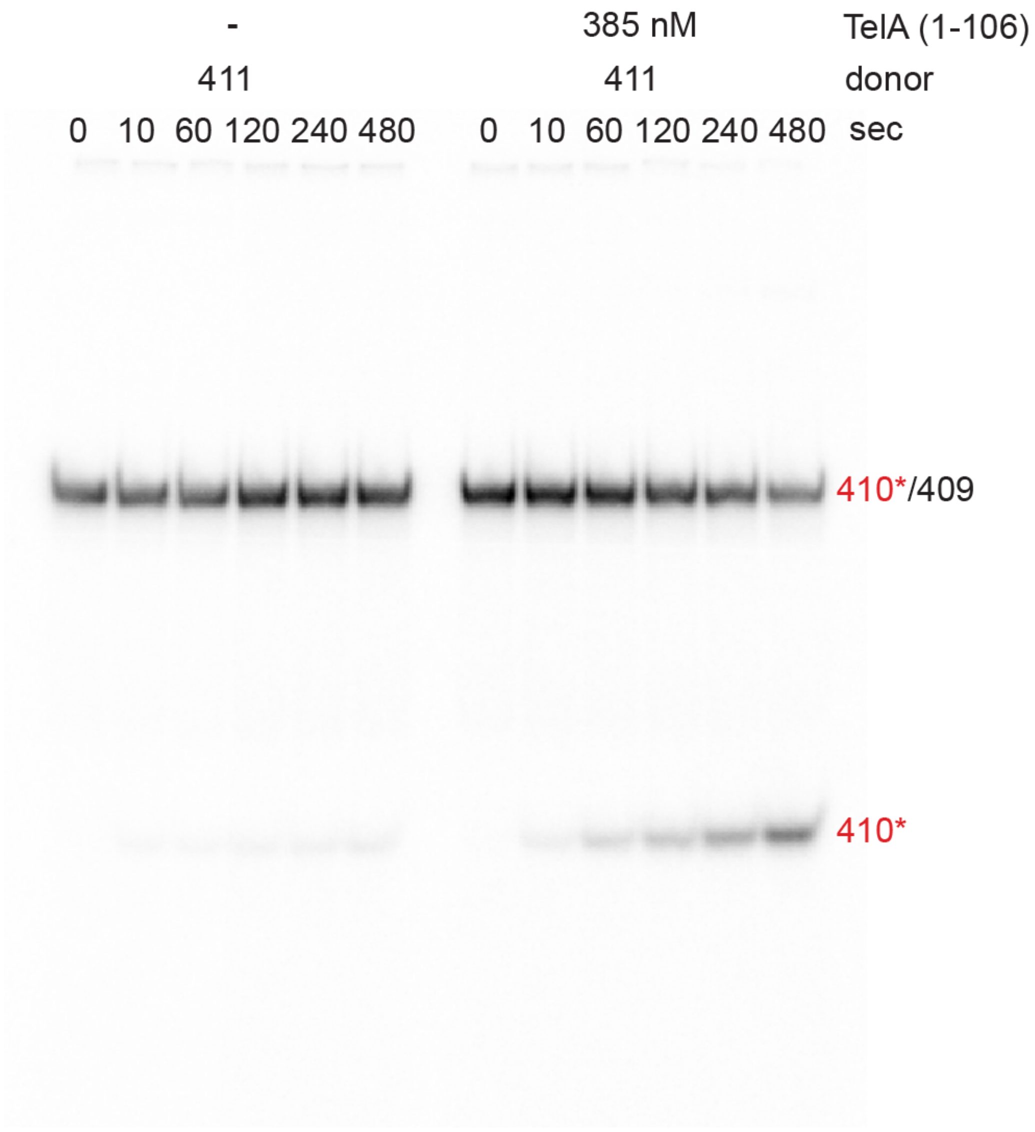

Gels for strand exchange assays shown in S6 Fig.
